# Selective ion permeation involves complexation with carboxylates and lysine in a model human sodium channel

**DOI:** 10.1371/journal.pcbi.1006398

**Published:** 2018-09-12

**Authors:** Emelie Flood, Céline Boiteux, Toby W. Allen

**Affiliations:** School of Science, RMIT University, Melbourne, Vic, Australia; University of Maryland School of Pharmacy, UNITED STATES

## Abstract

Bacterial and human voltage-gated sodium channels (Na_v_s) exhibit similar cation selectivity, despite their distinct EEEE and DEKA selectivity filter signature sequences. Recent high-resolution structures for bacterial Na_v_s have allowed us to learn about ion conduction mechanisms in these simpler homo-tetrameric channels, but our understanding of the function of their mammalian counterparts remains limited. To probe these conduction mechanisms, a model of the human Na_v_1.2 channel has been constructed by grafting residues of its selectivity filter and external vestibular region onto the bacterial Na_v_Rh channel with atomic-resolution structure. Multi-μs fully atomistic simulations capture long time-scale ion and protein movements associated with the permeation of Na^+^ and K^+^ ions, and their differences. We observe a Na^+^ ion knock-on conduction mechanism facilitated by low energy multi-carboxylate/multi-Na^+^ complexes, akin to the bacterial channels. These complexes involve both the DEKA and vestibular EEDD rings, acting to draw multiple Na^+^ into the selectivity filter and promote permeation. When the DEKA ring lysine is protonated, we observe that its ammonium group is actively participating in Na^+^ permeation, presuming the role of another ion. It participates in the formation of a stable complex involving carboxylates that collectively bind both Na^+^ and the Lys ammonium group in a high-field strength site, permitting pass-by translocation of Na^+^. In contrast, multiple K^+^ ion complexes with the DEKA and EEDD rings are disfavored by up to 8.3 kcal/mol, with the K^+^-lysine-carboxylate complex non-existent. As a result, lysine acts as an electrostatic plug that partially blocks the flow of K^+^ ions, which must instead wait for isomerization of lysine downward to clear the path for K^+^ passage. These distinct mechanisms give us insight into the nature of ion conduction and selectivity in human Na_v_ channels, while uncovering high field strength carboxylate binding complexes that define the more general phenomenon of Na^+^-selective ion transport in nature.

## Author summary

Ion channels can rapidly and selectively conduct an ionic species, essential for the firing of neurons, where sodium and potassium channels respond to changes in membrane potential to release stores of sodium and potassium ions in succession. The ability of a protein pore to discriminate between these two nearly identical ions has remained an intriguing problem for decades. In particular, the origins of sodium selectivity have been obscured by diverse protein chemistries that exhibit sodium-selective conduction in prokaryotes and eukaryotes. Here we use multi-microsecond atomistic simulations to observe and contrast the permeation mechanisms of sodium and potassium ions in model human and bacterial sodium channels. These channels exhibit shared features of conduction, centered on the involvement of charged protein groups that form complexes with the smaller sodium ion. In the human channel model, we observe a special role for the signature lysine that allows sodium to pass, but partially blocks potassium. As sodium channels are vital to heartbeat, sensation, muscle contraction and brain activity, these insights could assist developments in improved therapeutics for disorders such as epilepsy and chronic pain, with mechanisms relevant to a range of ion transport processes in biology and materials.

## Introduction

Voltage-gated sodium (Na_v_) channels are widely distributed in the central and peripheral nervous systems where they participate in essential functions, including heartbeat, muscle contraction and brain activity [1, 2]. Dysfunctional Na_v_s are associated with several physiological disorders, including epileptic seizures and chronic pain [3, 4], and they are therefore a major target for new drugs [5]. However, our understanding of the fundamental mechanisms governing these channels remains incomplete, largely due to the lack of high-resolution structural data on mammalian Na_v_s. There have, however, been recent breakthroughs in the structural determination for several Na_v_ channels that enable investigations into molecular mechanisms.

**Fig 1:**
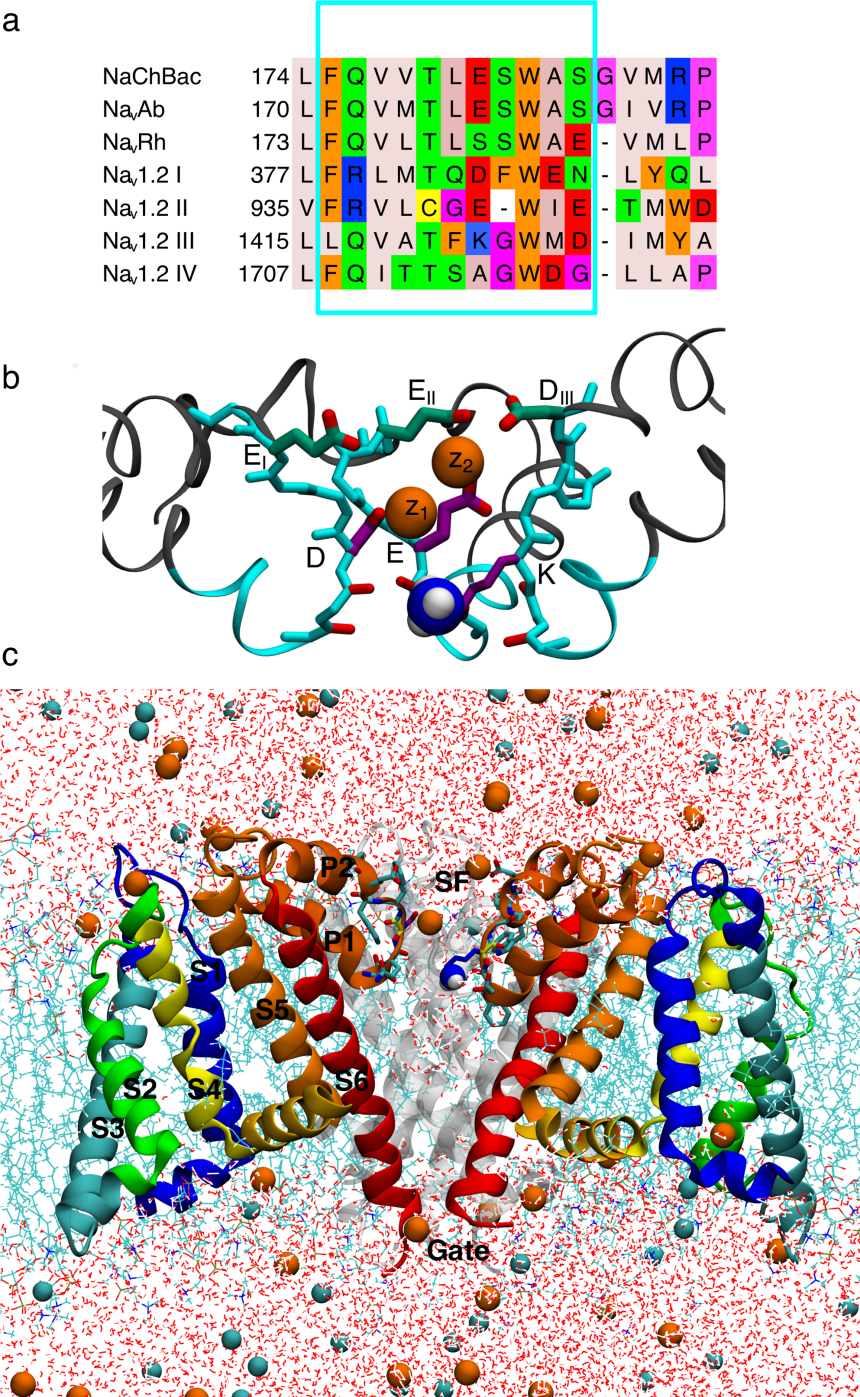
Human Na_v_1.2 model ion channel system. a) Jalview [81] sequence alignment of bacterial NaChBac, Na_v_Ab and Na_v_Rh, with the four domains of human Na_v_1.2 (labeled DI-DIV). Amino acids are colored according to their properties using the Zappo coloring scheme [81]. Cyan box selection marks the grafted Na_v_1.2 residues. Red box selection marks the EEEE/DEKA signature sequences responsible for selectivity. b) SF and vestibule of the Na_v_Rh/Na_v_1.2 model with grafted residues in cyan. The residues in the inner DEKA and outer EEDD rings are indicated in purple and green, respectively, and the charged ammonium group of the Lys is shown as blue and white balls. c) Na_v_Rh/Na_v_1.2 channel embedded in a hydrated DPPC bilayer (cyan sticks), surrounded by water (red and white sticks) with Na^+^ and Cl^-^ ions (orange and cyan balls). Three out of four monomers are shown (front subunit removed for clarity; rear subunit in gray), with VSD in green/blue/yellow and the PD in orange/red ribbons.

The first high-resolution structure of a Na_v_ channel was the X-ray structure of the bacterial channel Na_v_Ab [6], which was followed by numerous other high resolution structures, including for Na_v_Rh [7], Na_v_Aep1 [8] and Na_v_Ms [9, 10]. Although no mammalian Na_v_ structure is yet available, two eukaryotic Na_v_ structures have recently been solved using cryo EM; Na_v_PaS from cockroach [11] and a newly resolved EeNa_v_1.4 from electric eel [12], released after the simulations in this study were completed. Na_v_PaS has a 36–46% sequence identity to human Na_v_ channels, but is 300–500 residues shorter. Na_v_PaS has several residues in and around the SF unresolved, has reduced charge in the vestibule of the SF compared to human Na_v_s and a resolution of 3.8 Å [11]_._ EeNa_v_1.4 has a 65% sequence identity to the human Na_v_1.4 and a resolution of 4 Å [12]. The lower resolution of these structures is significant given the small size of the Na_v_ SF (diameter of ~3.6 Å at its narrowest point [12]), and thus while these new structures help guide and validate studies, there remains much to be learned from the existing high-resolution bacterial structures. Bacterial and human Na_v_ channels share several features including Na^+^-selective conduction, voltage-dependent activation, pore-based inactivation and drug modulation [13–16]. While there is only a 25–30% sequence identity between bacterial and human Na_v_s [10], there exists evidence for overall shared structure [13]. Human Na_v_s consist of four domains, DI-DIV, linked together to form one long polypeptide chain, whereas the simpler bacterial channels are made up of four identical subunits [13] (Fig 1A). Each of these domains/subunits consists of 6 helical trans-membrane spanning segments, S1-S6, where S1-S4 make up a voltage sensor domain (VSD) and S5-S6 the pore domain (PD). Between these two latter segments is a P-loop that includes a narrow ion selectivity filter (SF) [13] (Fig 1B and 1C), which establishes an ion preference “fingerprint” (permeability Li^+^~Na^+^ > K^+^~Cs^+^~Rb^+^) that is the same for both bacterial and human Na_v_s [1, 17, 18]. Both eukaryotic and bacterial Na_v_s are Na^+^ over K^+^ selective with eukaryotic Na_v_s selecting for Na^+^ with P_Na+_/P_K+_ ~10–30 [1, 19] and bacterial Na_v_s with P_Na+_/P_K+_ ~5–170 [17, 18, 20]. Due to the structural and functional similarities, the bacterial Na_v_ channels offer an excellent template, or scaffold, to support investigation into the core functional activities of mammalian Na_v_s.

Despite overall conservation of structure and function, the channels present distinct SF sequences (Fig 1A), with bacterial Na_v_ channels making use of a ring of four Glu side chains (EEEE), to create a high field strength binding site (S_HFS_) thought to favor Na^+^ [13], whereas all mammalian Na_v_ utilize a ring of Glu, Asp, Lys and Ala (DEKA; Fig 1A) [13, 19, 21], containing not only acidic residues, but also a basic lysine side chain that is known to be crucial to selectivity in mammalian channels [19, 21]. So how do these different channels achieve similar ion selectivity, and does the existence of disparate sequences mean that bacterial and mammalian channels impose their ion preference via different molecular strategies [18]? We seek to reveal and compare the fundamental rules of selective ion permeation for the whole family of sodium channels, by exploring ion conduction mechanisms in a human Na_v_ channel to compare to previous simulations in bacterial channels.

There are 9 different Na_v_s comprising Na_v_1.1 to Na_v_1.9 [22], with highly conserved sequences throughout these different subtypes [23]. Na_v_1.2 is abundant in the human nervous system and has been investigated extensively using methods of mutagenesis and electrophysiology (e.g. [21, 24, 25]). Here we take advantage of the conserved structural and functional features between mammalian and bacterial channels to generate a model of Na_v_1.2, which incorporates the key SF and vestibular regions, into a high-resolution bacterial structure. Such grafting has previously been successful experimentally for imparting Ca^2+^ selectivity on a bacterial Na_v_ channel [26]. The bacterial Na_v_Rh structure was chosen as the scaffold due to its higher sequence similarity in and around the SF (Fig 1A; only shown for Na_v_Ab, Na_v_Rh and Na_v_1.2, however, it can be seen that the same is true also for Na_v_Ms, Na_v_Ae and Na_v_Ct [16]). In particular, other bacterial Na_v_s have one residue extra just above the SF (Gly182; Na_v_Ab numbering) compared to Na_v_1.2 (Fig 1A). Furthermore, those channels have an arginine (Arg185; Na_v_Ab numbering) close to the SF that was found to interfere with the side chains of the Na_v_1.2 SF in our separate models using Na_v_Ab as a scaffold (not shown). We also note that proposed open structures of a bacterial Na_v_ have been presented [9, 16, 27], but our simulations (not reported here) have suggested that the proposed open Na_v_Ms structure does not stay open without strong constraints, as discovered previously [28]. Furthermore, previous studies of ion selectivity in bacterial Na_v_s using a closed lower gate have shown good sampling of ion translocation throughout the SF [18, 29–31], and are thus capable of shedding light on selectivity mechanisms by revealing the underlying equilibrium free energy surfaces governing multi-ion movements. There is minimal thermodynamic perturbation for ions in the SF and cavity due to the closed lower gate, with the free energy of an ion inside open and closed Na_v_Ab pores having been shown to be similar [27].

Previous simulations of bacterial channels have taught us much about the potential behavior of the human sodium channel. The bacterial SF contains a highly conserved ring of four Glu side chains forming a high field strength site (S_HFS_) [6]. Early simulation studies revealed ion binding sites and indications of a multi-ion conduction mechanism [28, 31–36]. These studies suggested favoring of a partly-hydrated Na^+^ due to the SF geometry in the Na_v_Ab crystal structure and the strength of interaction of ions with glutamate side chains [28, 31, 36]. They also demonstrated the ability of the SF to bind 2 ions concurrently within the S_HFS_ site, with higher affinity for Na^+^ potentially creating a reduced permeation barrier [36]. Simulations have shown that the SF is flexible and wide enough to house multiple Na^+^ ions [37], and is occupied by an average of two to three ions [29, 30, 38]; although simulations under significant membrane potentials have indicated reduced occupancy [28]. However, microsecond-length MD simulations have shown that the symmetric arrangement of Glu side chains in the crystal structure is broken on long time scales, affecting ion occupancy and having significant implications for the permeation mechanism [29, 30, 36, 39]. These studies demonstrated that there is coupling between ion translocation and SF conformation, with isomerization of glutamate side chains catalyzing Na^+^ conduction. These long unbiased simulations have described a stable 2-ion state, where the ions are trapped [30]. While some studies have focused only on this 2-ion state [31, 36], it has been shown that it is when a third ion enters the SF that efficient knock-on of the bottom ion into the central cavity occurs [18, 29, 30]. The top ion can either knock-on or pass-by the middle ion in S_HFS_, with both permeation pathways experiencing similar energy barriers (S2 Fig; [30]). Regardless, binding of Na^+^ to the S_HFS_ Glu side chains is central to understanding Na^+^ over K^+^ selectivity [18, 30, 31, 36].

Conduction is reliant on the flexibility of these side chains [29, 30, 36], where Na^+^ ions form favorable 2 ion-2 carboxylate clusters (see S2 Fig, states C_2_ and C_3_) that are not stable for K^+^ [30]. Human Na_v_ channels do not possess a symmetric E ring, but contain a set of carboxylates that might mediate similar complex formation. In particular, the DEKA signature sequence (Fig 1, right inset) contains two carboxylates that may be sufficient. However, human channels also possess a well-conserved [24] charged ring in the outer vestibule, immediately adjacent to the SF, consisting of Glu, Glu, Asp and Asp residues (E_I_E_II_D_III_D_IV_; with the exception of Na_v_1.7 that contains E_I_E_II_I_III_D_IV_) [40]. The residues in these two rings have been shown to be asymmetrical in position [41] and highly flexible [42], and thus may facilitate multi-ion conduction, akin to the bacterial channels.

Substitution experiments have shown that the DEKA-ring is crucial for the selection of Na^+^ ions in mammalian Na_v_ channels [19, 21, 25], with at least one of the D and E necessary to preserve selectivity [19]. The most important carboxylate in the DEKA ring is the E, which together with the K (residue 1422 in human Na_v_1.2), maintains wildtype (WT) selectivity, despite mutation of the D [19]. However, if the positions of these two residues (E and K) are swapped, into DKEA, selectivity is reduced nearly 4-fold [25], showing the importance of their precise locations, potentially due to interactions with surrounding amino acids, or implying a specific coordinating complex for Na^+^ during conduction. Mutagenesis has also shown that both the charge and length of the K_1422_ are necessary for maintaining Na^+^ selectivity; substitution of Lys with neutral side chains causing a complete loss of Na^+^ selectivity, and substitution with negatively charged side chains even reversing selectivity [19]. Previous computational studies examining the role of Lys in conduction have also been based on models using bacterial structures, and concluded on a passive blocking role for Lys. Xia et al. replaced the ring of four Ser in the bacterial channel Na_v_Rh with DEKA and performed MD simulations to find that only when the Lys was constrained to E_IV_ was the SF permeable to Na^+^ and K^+^ ions [43, 44]. Mahdavi et al. threaded the amino acids from rat Na_v_1.4 onto the backbone of a pore only model of Na_v_Ab, also suggesting that ion translocation requires displacement of Lys out of the permeation pathway, by hydrogen bonding to the neighboring DIV Ser [45]. We will show in this study, using extended length simulations, that Lys in fact plays an active role in conduction (not just based on pore occlusion) that is quite different for Na^+^ and K^+^ ions.

The vestibular EEDD-ring has also been shown to be important for ion conduction, possibly because it increases the electrostatic attraction of extracellular cations [24, 25, 46, 47]. However, all four residues are not equally important, with cysteine mutations showing the greatest decrease in conduction occurs when domain II Glu is mutated (effect on conduction: E_II_>E_I_> D_IV_~D_III_) [25, 41]. Furthermore, mutagenesis experiments have shown that replacement of EEDD residues can be detrimental to Na^+^ selectivity. In particular *P*_*Na*_/*P*_*K*_ has been shown to decrease when D_IV_ is replaced with Cys [40, 41, 48], while other experiments have shown little or no effect [25, 49]. Functionally, therefore, the exact roles of the outer ring carboxylates in selectivity are not well defined. Previous models based on bacterial structures have implied a greater thermodynamic preference for Na^+^ in the lower SF, and an additional outer binding site at the EEDD-ring [43, 45]. However, we will show that on long timescales, SF rearrangements allow for stable complexes with carboxylates from both the S_HFS_ and outer ring that are important to Na^+^ permeation.

Experiments have suggested that Na_v_s possess multiple binding sites inside the SF [50–53]. However, the evidence for their ability to bind multiple ions at the same time is inconclusive [50–55]. Investigations of flux coupling suggest a flux ratio exponent close to unity for mammalian Na_v_ channels, which is generally thought to imply a 1-ion conduction mechanism [51, 54, 55]. Furthermore, anomalous mole fraction effects, considered evidence of ion cooperation during conduction, have only been observed in bacterial [18] and not mammalian Na_v_s [53]. Such effects are normally expected in single-file channels that impose ion correlation [56], but may also arise from preferential and localized ion binding [57–59]. Single-file conduction is unlikely in Na_v_ channels given their size [60] and flexibility [42]. However, absence of an anomalous mole fraction effect does not rule out ion cooperativity. The fact that anomalous mole fraction effects are seen in bacterial Na_v_ channels in the absence of bona fide single-file permeation, as suggested by several simulation studies, as well as the decoupling of ion and water fluxes [28], demonstrates limitations in the ability of this measure to identify a multi-ion mechanism. Likewise, there exists contradictory evidence from single channel conductance measurements for mammalian Na_v_ channels, which have shown a saturating dependence on concentration in some experiments, indicative of a single ion mechanism [50–52], whereas others, where a wider range of concentrations have been examined, witness a complex relationship that may be explained by a multi-ion conduction mechanism [53]. Thus the experimental evidence is inconclusive when it comes to the ion occupancy and potential multi-ion conduction mechanism in mammalian Na_v_s. Molecular dynamics (MD) simulations can therefore help us elucidate the mechanisms involved in ion selectivity in Na_v_s.

Bacterial and human Na_v_ channels therefore both possess several carboxylate side chains in (or adjacent to) the SF, allowing for multiple high field strength sites that may bind one or more ions, possibly leading to increased Na^+^ selectivity [61]. In a multi-ion/multi-ligand complex there will be competition between favorable ion-ligand and unfavorable ligand-ligand and ion-ion energies [62], needing long time scale simulations to capture the ion and protein configurations [29, 30]. The mechanism is further complicated by the presence of the Lys, which may increase the sampling challenges due to side chain isomerizations. We thus turn to long MD simulations to capture those ion and slowly interconverting protein movements using the DE Shaw Anton supercomputer. We have performed multi-μs simulations where Na^+^ and K^+^ are permitted to freely diffuse into and out of the SF of the Na_v_Rh/Na_v_1.2 model, observing the involvement of DEKA and outer EEDD rings, as well as participation of the signature Lys in multi-ion binding complexes, revealing distinct permeation mechanisms for Na^+^ and K^+^ ions.

## Methods

We have created a model of human Na_v_1.2 that includes the most important residues for ion permeation and selectivity, using a structurally well-defined bacterial channel as a scaffold. Although other parts of the channel may be important for attracting ions [11], the main functional region involves the SF and outer vestibular regions [24]. The sequence alignment in Fig 1 was used to create a model of human Na_v_1.2 based on the bacterial Na_v_Rh structure (PDB:4DXW [7]), due to their higher sequence homology in and around the SF region, as discussed above. The mutated region (framed in cyan in Fig 1A), spanning residues 174–184 (Na_v_Rh numbering used herein), was chosen to introduce the SF and vestibular sequence with minimal perturbation. This includes the human DEKA and EEDD rings (residues 180 and 183/184) in the upper SF. Furthermore, residues 174–177 are located behind the SF and may interact with the side chains of the SF. In particular, within that range is Na_v_Rh Q175, which is a positively charged Arg in Na_v_1.2 DI and DII, and may influence both structure and ion conduction. To minimize perturbation, side chains of the chosen amino acids were removed from Na_v_Rh and the corresponding Na_v_1.2 side chains rebuilt manually using the CHARMM program [63]. Herein the residues from the DEKA ring will be referred to by their 1-letter abbreviations. To avoid confusion with the symbol for Lys (K), potassium will always be referred to in ionic form, K^+^. The Asp and Glu residues from the vestibular EEDD ring will be referred to according to their domain numbers (E_I_, E_II_, D_III_ and D_IV_).

Between the DEKA ring and the EEDD ring the Na_v_1.2 sequence is one residue shorter in DII than in the bacterial channels. This residue (number 181) was removed, and neighboring residues (180–182) joined with constrained MD simulation (see below). We note that the original Na_v_Rh SF structure was closed at the lower gate as well as having a slightly collapsed SF. The collapsed SF has been proposed to be associated with the orientation of the Ser181 side chains, blocking the SF [64], however, after removing the Ser side chains as well as patching Na_v_1.2 residues and equilibration (described below), the filter forms an open SF conformation that allows us to study ion permeation (Fig 1B and 1C). Other changes to the model, including the rebuilding of missing intracellular loops connecting S2 and S3 helices in the VSD using Rosetta [65], and the maintenance of a key hydrogen bond between Thr178 and Trp182 that has been postulated to be important for keeping the shape of the SF in bacterial Na_v_ [6, 7], are described in the Supplementary Information.

It is difficult to predict or measure the pK_a_ shift of Lys in a non-aqueous microenvironment with the coming and going of conducting ions, such as in the SF of a Na_v_ channel [66]. Large pK_a_ shifts have been recorded in channel environments, and protonation states depend on the exact environment and ion occupancy, which will fluctuate over time [67]. The dependence of Na_v_ conduction on the protonation states of key residues in the SF has therefore been investigated in several studies of bacterial Na_v_s [30, 36, 68]. Furthermore, quantum mechanical calculations using simplified models of the DEKA ring alone have demonstrated that a thermodynamic preference for Na^+^ may be achieved with either protonation state of the Lys [61]. However, the signature Lys has been shown to be crucial to Na^+^ selectivity [19], intuitively suggesting that its charge may be important. To cover both possibilities, we have examined Na^+^ ion movements with both charged and neutral lysine, which will help us isolate the role of that charge in ion conduction.

The proteins were embedded in lipid bilayers of dipalmitoyl-phosphatidylcholine (DPPC), being the best characterized lipid for MD simulations [69], with explicit TIP3P water molecules [70] and 150 mM of NaCl or KCl solution. Systems were built and pre-equilibrated with CHARMM [71] and further equilibrated using NAMD [72] prior to unbiased production simulations carried out on the purpose-built supercomputer Anton [73, 74] for 4 *μs* for Na_v_Rh/Na_v_1.2 with charged Lys both for NaCl and KCl, and 2 *μs* for neutral Lys (less time required due to better sampling of ion movements), totaling 10 *μs*. Simulations all used the CHARMM36 [69] lipid and CHARMM22 protein and ion parameters [72] with CMAP corrections [75], chosen to provide direct comparison to our past simulations of the bacterial Na_v_Ab channel [30]. However, attention to ion-carboxylate parameters was given to ensure accurate interactions for Na^+^ and K^+^ inside the SF, with standard parameters for the ion-carboxylate interaction shown to lead to reasonable agreement with both *ab initio* MD free energies of binding and osmotic pressure data [76]. Descriptions of both ion-carboxylate and corrected ion-carbonyl interactions are discussed in the Supplementary Information.

The free energy map, or potential of mean force (PMF), for ion movement was calculated for each system using trajectory data with specific pore ion occupancies, from unbiased simulations as *W*({*z*_*i*_}) = −*k*_*B*_*T* ln *ρ*({*z*_*i*_}) + *C*, where *ρ* is the unbiased probability distribution as a function of reaction coordinate(s) {*z*_*i*_}, being the vertical positions of one or more ions (*z*_1_, *z*_2_, or *z*_3_; defined with *z*_1_ being the lower-most ion), or their centroids (e.g. *z*_12_ = (*z*_1_ + *z*_2_)/2), each relative to the center of mass of the protein, *z*_*ref*_, and where *C* is a constant. See Supplementary Information for more details, including error calculations.

The most important states involved in the permeation of Na^+^ and K^+^ ions across the SF were identified from the PMFs and cluster analysis. The frames were broken down according to ion occupancy as well as ion-ion and ion-carboxylate distances (S1 Table). Different states were identified according to how many carboxylates were interacting with the ion/ions using a cutoff defined from the radial distribution functions in S3 Fig, with further details of the clustering method described in the Supplementary Information. These states include complexes involving one or more carboxylate groups, Na^+^ or K^+^ ions and/or the signature Lys ammonium group. To evaluate the relative binding affinities of these states, Free Energy Perturbation (FEP) [77] calculations were performed to obtain the relative binding free energies of Na^+^ and K^+^ to each particular complex. Details of these, and other calculations, including structural comparisons of available X-ray and Cryo-Em structures, and continuum electrostatic calculations to examine ion-binding propensities, are provided in the Supplementary Information.

## Results and discussion

### A stable human Na_v_ model with asymmetric and flexible selectivity filter

After patching of Na_v_1.2 SF and vestibular residues into the bacterial channel, followed by equilibration, the overall structures of the PD and SF were stable and settled down to RMSD 2–3 Å (S4 Fig) after initial changes during the first *μ*s of simulation, with the level dependent on the choice of Lys protonation state (see below). Late changes are seen, especially for simulations in KCl, due to the onset of permeation following conformational changes within the SF, described below. The first 1 *μ*s of the Na_v_1.2 model with charged Lys with NaCl and KCl and the first 0.25 *μ*s of the shorter simulation with uncharged Lys have been discarded as equilibration (S5 Fig). We observe that the Na_v_1.2 SF is relaxing into an asymmetrical shape where the DEKA and EEDD-ring span ~10–15 Å, allowing interactions with residues other than their immediate neighbors (S5 Fig), explaining the increased RMSD. Previous experiments involving cysteine mutations have shown that the residues in the filter of an Na_v_ channel are indeed asymmetrical in height and that the inner and outer rings span up to ~15 Å [41]. This asymmetry destabilizes the Thr/Cys178-Trp182 bond, important for keeping the shape of the SF in bacterial channels [6, 7], which are seen to break and form several times during the simulations.

Flexibility has been shown to be a critical property of the bacterial Na_v_ channel SF to facilitate ion conduction [29, 30]. Furthermore, pairwise cysteine mutations have suggested significant flexibility (up to 7 Å) of the residues in the SF of a mammalian Na_v_ [48] [42]. We observe high flexibility in the Na_v_Rh/Na_v_1.2 SF (S6 Fig, with mean SF backbone fluctuations being approximately twice as large as those for Na_v_Ab (RMSF~1.5 Å and RMSF~0.8 Å for Na_v_Rh/Na_v_1.2 and Na_v_Ab, respectively) (S6A and S6B Fig). These RMSF values (S6A Fig) are mostly due to asymmetric subunit movements, with the RMSF values halved when computed based on subunit-by-subunit orienting (S6B Fig).

The recent eukaryotic Na_v_PaS [11] and EeNa_v_1.4 [12] cryo EM structures offer related structural data to validate our model, although at limited resolution where several SF side chains were not resolved, preventing immediate conclusions about specific interactions. Structural alignment between the Na_v_Rh/Na_v_1.2 model and the Na_v_PaS or EeNa_v_1.4 structures (S1 Fig) shows structural similarities between the proteins, with key residues at the same positions. Due to the highly dynamic nature of the SF and its side chains, structural comparisons are limited and will depend on the choice of structure from the model simulation. Overall, however, structures are consistent, with the RMSD of the SF and vestibular region (residue 178 to 184) backbone being 2.0 Å and 2.3 Å when aligning to all four subunits (S1A Fig), and 1.8 Å and 2.1 Å when aligning subunit by subunit (S1A Fig), for Na_v_PaS and EeNa_v_1.4, respectively. The residues of the DEKA and EEQD/EEDD rings are asymmetrical in position in all three structures, with these residues spanning 10–15 Å in the model, ~10 Å in Na_v_PaS and ~14 Å in EeNa_v_1.4 (S1 Fig). The spread of these side chains in both our model and the Na_v_PaS and EeNa_v_1.4 structures, combined with the high flexibility and lack of experimental resolution in this region, indicates that SF dynamics may play an important role in conduction.

### Selectivity filter with neutral lysine exhibits bacterial-like multi-ion complex formation and conduction

The DEKA Lys has been shown to be crucial to Na^+^ selectivity in mammalian Na_v_ channels [19]. However, the protonation states of amino acids in a dynamic microenvironment, like that seen here within the SF), are hard to pinpoint experimentally [66] and may have large fluctuations [67]. We have therefore investigated ion permeation in Na_v_1.2 not only with a protonated (Lys^+^) but first also with a deprotonated Lys (Lys^0^). Importantly, this allows us to isolate the role that the positive charge may play in the SF of Na_v_1.2 for Na^+^ permeation, and offers a comparison to existing bacterial Na_v_ simulations.

**Fig 2:**
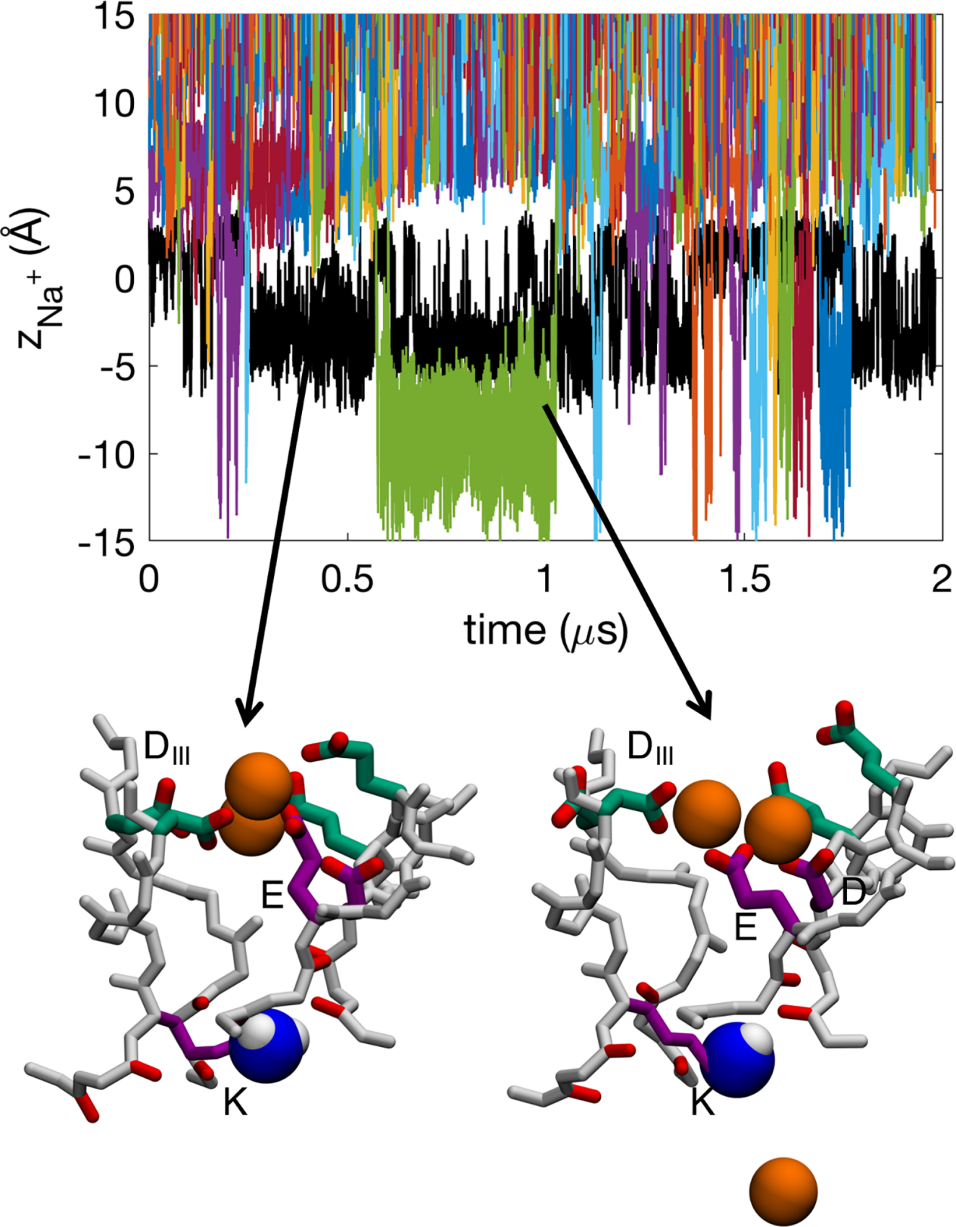
Time series showing the *z* positions of deprotonated Lys^0^ (K; black) and Na^+^ ions (colors) in the Na_v_1.2 SF. Insets show snapshots of Na^+^ ions (orange balls) in representative configurations with active residues from the DEKA (purple) and EEDD (green) rings labeled. The neutral amine group of the Lys shown is shown as blue and white balls. See also sample trajectory in **S1** Movie.

**Fig 3:**
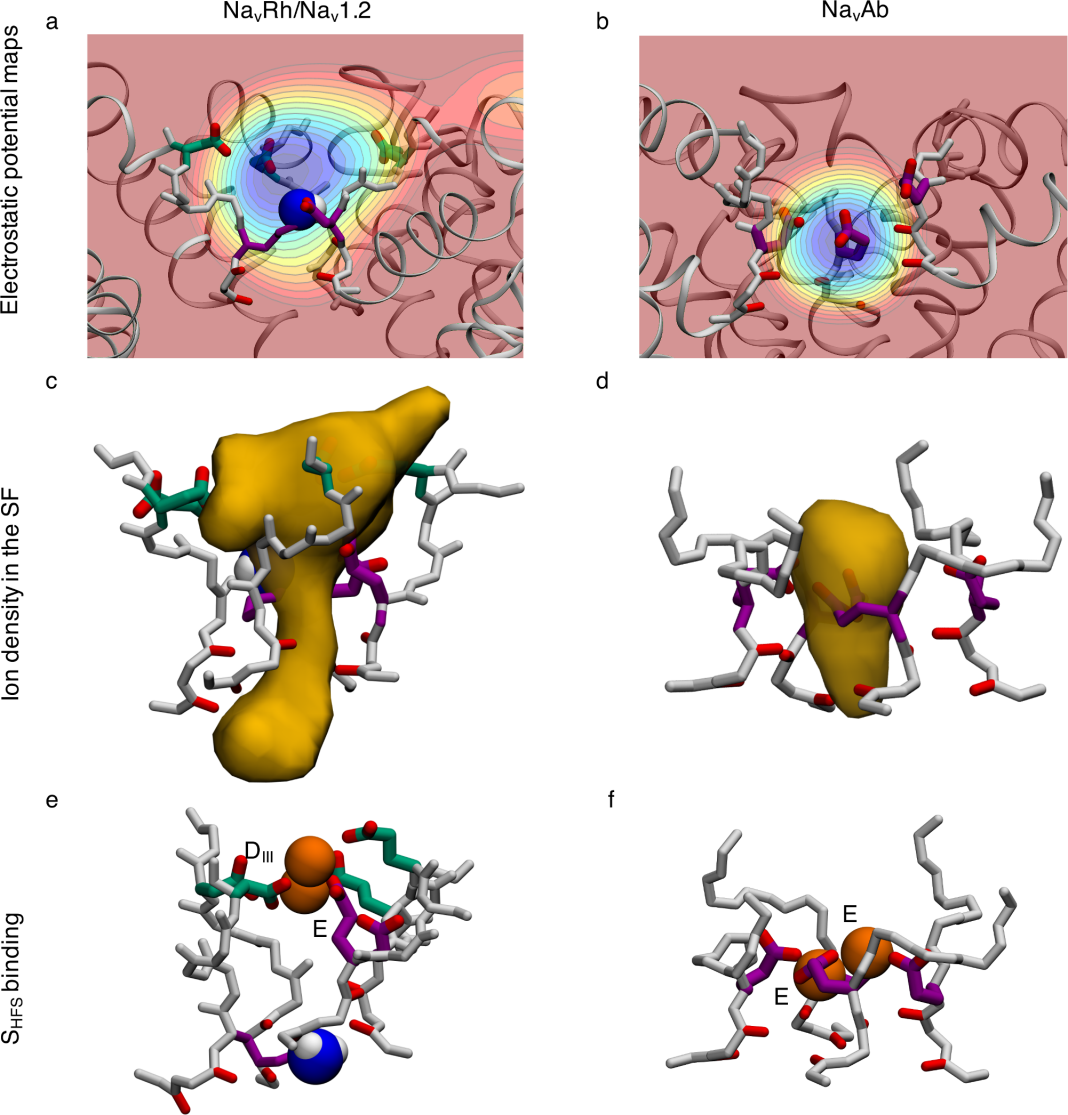
Comparison of electrostatic potential maps. Electrostatic maps of a) the Na_v_1.2 SF with deprotonated Lys^0^, compared to b) bacterial Na_v_Ab. Each contour represents 10 kcal/mol/Å/e. Density maps of Na^+^ occupancy in c) the Na_v_1.2 SF with Lys^0^ and d) Na_v_Ab. Multi ion-multi carboxylate binding sites in e) Na_v_1.2 and f) Na_v_Ab. The residues in the inner DEKA and outer EEDD rings are indicated in purple and green, respectively, and the uncharged amine group of the Lys is shown as blue and white balls.

Permeation of Na^+^ in the SF of the Na_v_1.2 model when its DEKA Lys is neutral involves both 2-ion and 3-ion conduction mechanisms with an average number of 2.1±0.2 Na^+^ ions in the SF, similar to (albeit slightly less than, though within the errors) that seen in the bacterial channel Na_v_Ab (2.3±0.5 ions; [30]). The ions are partly hydrated as they permeate the SF (S7 Fig). In the time series for ion movements shown in Fig 2, several ions (colored lines) are observed to enter and exit the central cavity (below −5 Å), often coexisting within the SF (S1 Movie). We see 61 complete permeation events through the SF (inward or outward moving involving 16 distinct ions) and the ions appear to move independently of the neutral Lys side chain (black line). Snapshots in Fig 2 show representative configurations of a 2-ion and 3-ion state, where we see multi Na^+^-ion/multi-carboxylate complexes similar to those in for the bacterial Na_v_ ([30]; see also Fig 3E and 3F, to be discussed below). However, in the Na_v_1.2 channel it most commonly not only involves carboxylates only from the inner DEKA-ring, but also from the outer EEDD-ring, whose carboxylates reach down to help coordinate the ions, as opposed to only using two carboxylates from the inner ring, as was the case of the bacterial Na_v_Ab channel [30].

This cooperation between the DEKA and EEDD-rings creates a longer SF binding region with a broad span of negative electrostatic potential (Fig 3A and 3B), leading to less distinct binding sites. While Na_v_Ab has all of its charged side chains in one symmetrical ring, Na_v_1.2 has its charges spread out in two asymmetrical and highly flexible rings creating a broad S_HFS_ (ion density map shown in Fig 3C), compared to Na_v_Ab (Fig 3D). Commonly a binding site is created with two Na^+^ and one or two carboxylates from the DEKA and EEDD rings (S1 Table; column 2 and S8 Fig). The specific residues involved vary and we see several distinct carboxylate-ion complexes forming during the simulation (S1 Table; column 2 and S8 Fig).

Double ion occupancy dominates in the SF, but 3-ion occupancy is also common (S8A Fig). When the SF is occupied by a single ion it is most commonly unbound, however, it is also likely to be bound to one or more carboxylate side chains (S8B Fig). When there are two ions in the filter they most likely form single ion complexes with multiple carboxylates (S8C Fig). They are also often singly bound or bound together in a tight multi-ion/multi-carboxylate complex (defined by the radial distribution function in S3 Fig; with ions within 4.7 Å of each other and 2 or more carboxylate groups (based on central C atom) from the DEKA and EEDD-rings within 3.8 Å of the ions). When there are three ions in the SF, two of these are most likely to be bound together in a tight multi-carboxylate complex (S8D Fig). We have previously shown that multi-ion/multi-carboxylate complexes play a role in Na^+^ selectivity in acid sensing ion channels [76]. When these complexes are present, the ions are mostly bound by E and/or D from the DEKA ring, together with the outer ring D_III_ (S1 Table; column 2). This cooperativity of inner and outer charge rings is common and while all carboxylate side chains from either of the two rings ring are not necessary, at least one is needed for Na^+^ binding in the S_HFS_. In particular, the DEKA ring is involved ~80% of the time, the EEDD ring ~80% of the time, and both rings cooperatively involved ~60% of the time. This need for only one of the carboxylates in the DEKA ring to maintain cation permeability has previously been implicated by mutagenesis experiments [19].

**Fig 4:**
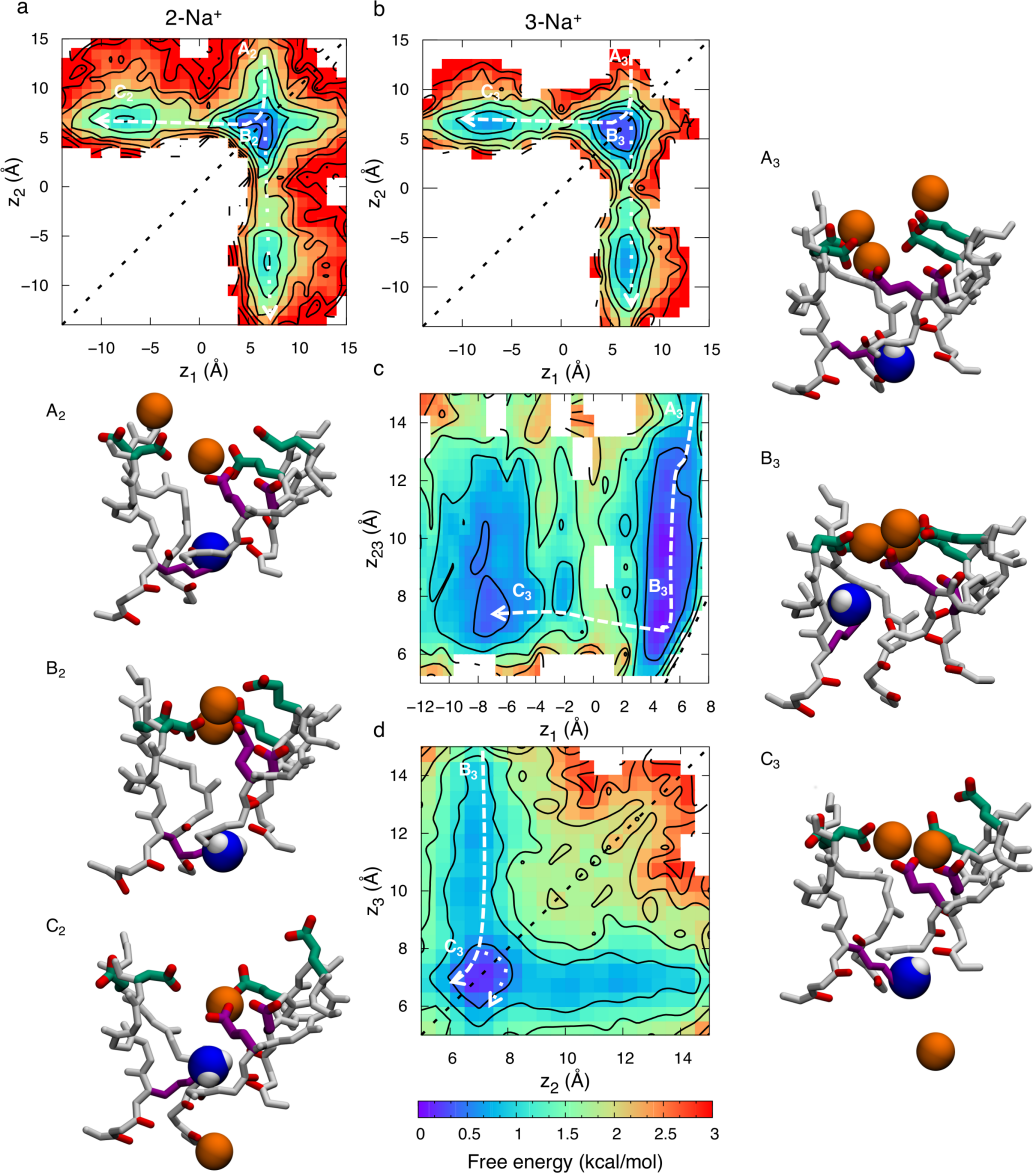
Permeation of Na^+^ in the Na_v_1.2 SF with deprotonated Lys^0^. 2D free energy projections showing a) 2-ion conduction mechanism, and b, c & d) 3-ion conduction mechanism (graphed for the two bottom ions in b, all three ions in c, and the two top ions in d); where *z*_1_, *z*_2_, and *z*_3_ correspond to the *z* positions of the bottom, middle and top ions, respectively, and where *z*_23_ is the *z* position of the COM of the top two ions. Each contour represents 0.5 kcal/mol. Snapshots, with the inner DEKA ring in purple, the outer EEDD-ring in green, and the uncharged amine group of the Lys shown as blue and white balls, indicate the corresponding Na^+^ ion (orange ball) movements. State labels (A-D) include a subscript 2 or 3, representing the 2-ion or 3-ion mechanisms, respectively.

Despite the longer SF binding region in Na_v_1.2 with neutral Lys, we see a conduction mechanism resembling that of the bacterial Na_v,_ relying on multi ion-multi carboxylate clusters for an efficient knock-on conduction. When there are 2 ions within the SF, at *z*_1_ (lower) and *z*_2_ (upper), the free energy map (as function of *z*_1_ and *z*_2_) in Fig 4A exhibits three states; A_2_, B_2_ and C_2_. In state A_2_, the upper ion is in the vestibular region of the SF (*z*_2_~14Å) and the lower ion is collectively bound by the inner (purple side chains) and outer rings (green side chains) (*z*_1_~7Å). In state B_2_ the top ion has joined the bottom ion and they are both bound by the inner and outer rings (*z*_1_~*z*_2_~7Å) forming cooperative tight multi-ion/multi-carboxylate clusters, commonly made up by D, E and D_III_, and reminiscent of those seen in the bacterial Na_v_ (compare Fig 3E and 3F). In state C_2_ the top ion has either pushed the bottom ion into the cavity by Coulomb repulsion (dashed line) or it has passed by the bottom ion and entered the cavity itself (dotted line crossing the diagonal, y = x, represented by black dashed line), in both cases translocating from *z*_2_~7 Å to *z*_2_~-10 Å, while the other ion remains collectively bound to the inner and outer rings. These distinct permeation pathways are equally likely, with the largest barrier encountered being 1.9±0.8 kcal/mol.

Fig 4B–4D show free energy projections for the 3-ion occupancy state, with ion positions specified as *z*_1_ (lower ion), *z*_2_ (middle ion), *z*_3_ (upper ion) or *z*_23_ (COM of the two upper ions). Here state A_3_ has one ion in the middle of the two rings (*z*_1_~7 Å) and one ion in the vestibule (*z*_2_~14 Å), and an additional ion entering from the bulk above (*z*_3_~15 Å). In Fig 4B and 4C we see how the upper ion pushes the middle ion downward to bind together with the lower ion at the inner ring (*z*_1_~*z*_2_~6 Å), represented by state B_3_, before eventually entering this site itself and pushing the bottom ion into the cavity, state C_3_, completing the conduction event. The broad free energy surface in panel c is due to the range of binding sites offered by the inner and outer carboxylate rings, as well as the fact that the centroid of 2 ions may span a wide range. Fig 4D shows another projection involving only the top two ions (*z*_2_ and *z*_3_). The dashed line in Fig 4D shows how the top ion moves from state B_3_ to state C_3_ by entering the S_HFS_ and knocking the bottom ion downward. The dotted line shows an alternative path where the top ion instead passes by the middle ion before knocking the bottom ion into the cavity. Regardless of the permeation pathway, the largest barrier experienced during permeation through the SF in the 3-ion state is 1.4±0.6 kcal/mol, similar to that experienced for Na^+^ in the bacterial channel (S2 Fig). These results demonstrate that low barrier conduction may occur via knock-on or pass-by mechanisms in either 2- or 3-ion occupancy states for Na_v_1.2, but where 3-ion conduction is energetically more favorable, with reduced activation barrier. However, considering a 2-ion occupancy is more common (S8 Fig), the 2-ion mechanism is likely also contributing significantly to the overall ion flux.

We therefore have observed that the mammalian SF conducts Na^+^ ions well with a neutral/deprotonated Lys in the DEKA ring, providing a useful comparison to the bacterial channel, with similar multi-ion/multi-carboxylate complexes forming during permeation (although involving both SF DEKA and vestibular EEDD carboxylates). However, because Lys is so important to permeation and selectivity experimentally [19, 21, 25], but when neutral apparently plays only a passive role that would not implicate it, this suggests that we must turn our attention to the protonated Lys case.

### Charged lysine forms a high field-strength complex to facilitate Na^+^ conduction

**Fig 5:**
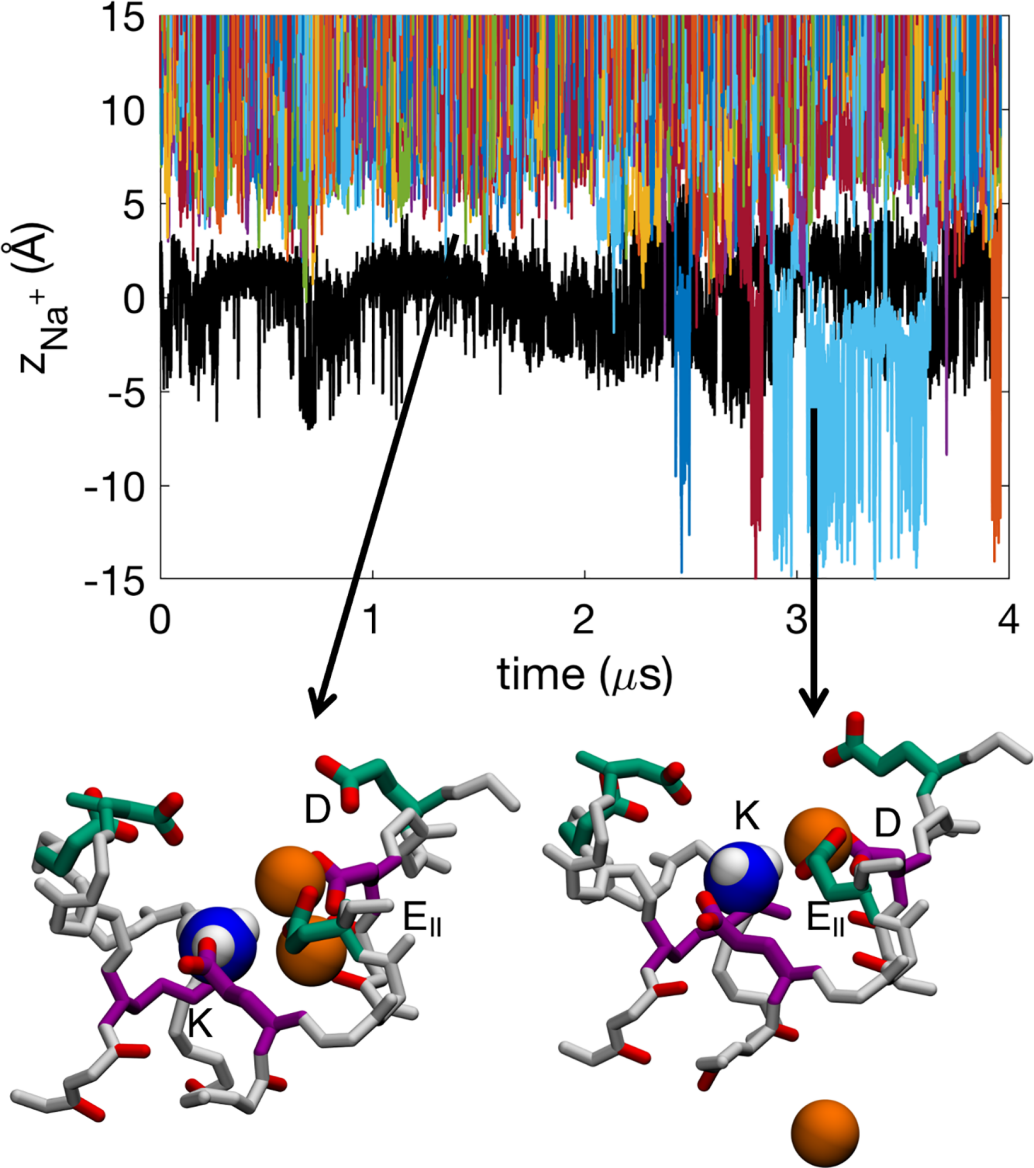
Time series of *z* positions for protonated Lys^+^ (K; black) and Na^+^ ions (colors) in the Na_v_1.2 SF. Insets show snapshots of Na^+^ ions (orange balls) in representative configurations with active residues from the inner DEKA (purple) and outer EEDD (green) rings labeled. The charged ammonium group of the Lys is shown as blue and white balls. See also sample trajectory in **S2** Movie.

When Lys is charged, there is an average of 1.5±0.4 Na^+^ ions in the SF and permeation involves either 1 or 2-ion conduction (S2 Movie). This reduction in ion occupancy (compared to the neutral Lys case above and the bacterial channel) is presumed to be due to the reduced negative charge inside the SF. If the ammonium group of the Lys was considered as another ion, we would have, in total, a similar ion occupancy of 2–3 ions in the SF. (n.b. Lys^+^ may be considered another ion, as Na_v_ channels conduct ammonium ions nearly as well as Na^+^, with relative permeability P_NH4+_/P_Na+_ = 0.16 [60]). The time series in Fig 5 reveals that several ions (colored lines) enter and leave the SF, but it is not until the second half of the 4 μs simulation that ions begin to cross the charged Lys and enter the cavity beneath, after which we observe 28 complete permeation events (defined as any ion crossing downward or upward past the Lys, and involving 6 distinct ions that experience repeat crossings).

Snapshots in Fig 5 show representative configurations of 1- and 2-ion carboxylate complexes. As with the neutral Lys case, we see multi-ion/multi-carboxylate complex formation where the residues from the EEDD ring (green) bend down into the SF region, leading to the binding of ions together with the carboxylates from the DEKA ring (purple; see also S1 Table; column 3). The DEKA ring is involved in binding ~50% of the time, the EEDD-ring ~90% of the time, and both rings cooperatively involved ~40% of the time. These complexes are most commonly (69±11%) involving D from DEKA and E_II_ from EEDD. The ammonium group of the Lys from DEKA is also involved, binding 61±10% of the time to the E or D from the DEKA-ring. The salt bridge between Lys and one or both of these residues has previously been suggested by mutagenesis experiments as being important for Na^+^ selectivity [19], and may be responsible for the importance of the precise sequence position of the Lys in the DEKA ring [25]. We also observe that when ions enter the SF, the ammonium group of Lys (black line in Fig 5) is displaced downward; being knocked-on, like any other ion. Thus, the charged Lys is intimately involved in the multi-ion mechanism. Importantly, it also participates in a Lys+Na^+^/carboxylate complex, similar to the 2-ion/multi-carboxylate clusters, commonly coordinated by D and E_II_ and sometimes additionally by E (S1 Table; column 5). The ability to form these complexes is likely dependent on the location of the Glu and Lys in the DEKA ring, and swapping their positions might affect this complex formation thus decreasing selectivity, as indicated by mutagenesis experiments [25]. We will see below how this tight cluster plays an important role in the conduction mechanism.

**Fig 6:**
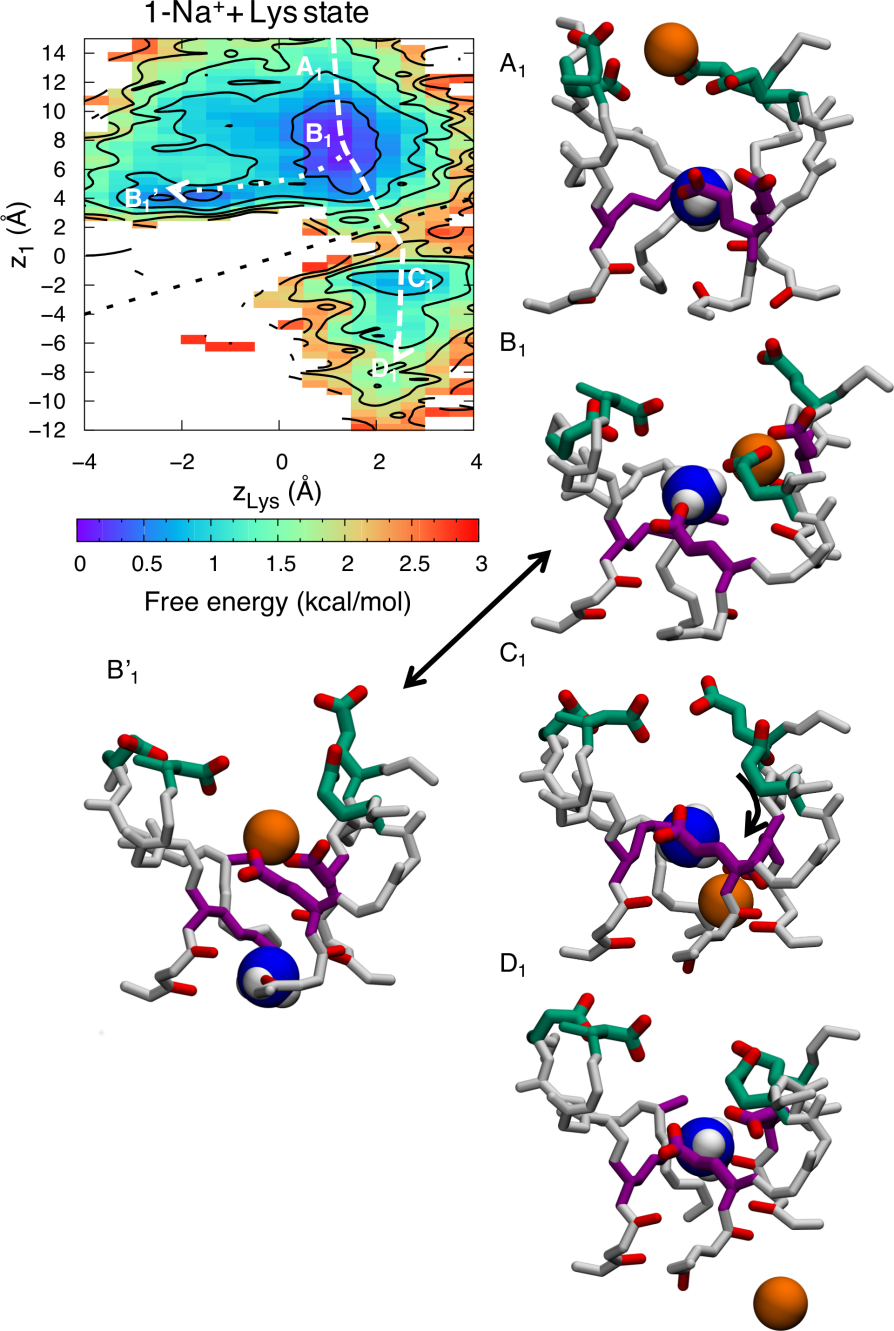
Single ion permeation for Na^+^ in the Na_v_1.2 SF with protonated Lys^+^. 2D free energy projections showing 1-ion conduction mechanism where *z*_1_ corresponds to the *z* position of the ion and *z*_Lys_ the *z* position of the Lys^+^ ammonium group. Snapshots, with the inner DEKA ring in purple, the EEDD ring in green and the charged ammonium group of the Lys shown as blue and white balls, indicate the corresponding Na^+^ ion (orange balls) movements.

Free energy projections reveal the relationship between the ions and the position of the ammonium group of Lys, *z*_Lys_, demonstrating conduction with 1-ion and 2-ion occupancies in the SF. When there is a lone ion in the SF, at *z*_1_, the free energy map (as function of *z*_1_ and *z*_Lys_) in Fig 6 exhibits four states; A_1_, B_1_, C_1_ and D_1._ In state A_1_, the ion is in the outer vestibular region of the SF (*z*_1_~14Å) and the Lys is bound to one of the inner ring carboxylates (*z*_Lys_~1 Å). In state B_1_, the ion has entered deeper into the SF and is bound to the inner and outer rings (*z*_1_~6 Å). The dotted line shows an alternative state, B_1_’, in which the ion entering the filter replaces the ammonium group of the Lys, which is displaced downward (from *z*_1_~6 Å & *z*_Lys_~1 Å to *z*_1_~4 Å & *z*_Lys_~-2 Å). It is interesting to note that there is no conduction occurring when this happens. The ion is bound tightly to the DEKA ring and the ammonium group of the Lys is too repulsive for it to pass by in the lower part of the filter, where the electrostatic potential is not as negative. However, the dashed line shows how the ion can cross the ammonium group of the Lys in a region of higher field strength created by multiple carboxylates (*z*~2 Å), leading to state C_1_. In state C_1_ the ammonium group of the Lys remains bound to the inner ring (*z*_Lys_~2 Å) and the ion is bound to the backbone carbonyl of the two residues underneath the DEKA ring (*z*_1_~-2 Å). In state D_1_ the ion has left the SF and entered the cavity (*z*_1_~-8 Å), completing the permeation event. The dashed arrow shows the minimum free energy path for conduction, where the largest barrier encountered is 1.7±0.3 kcal/mol.

**Fig 7:**
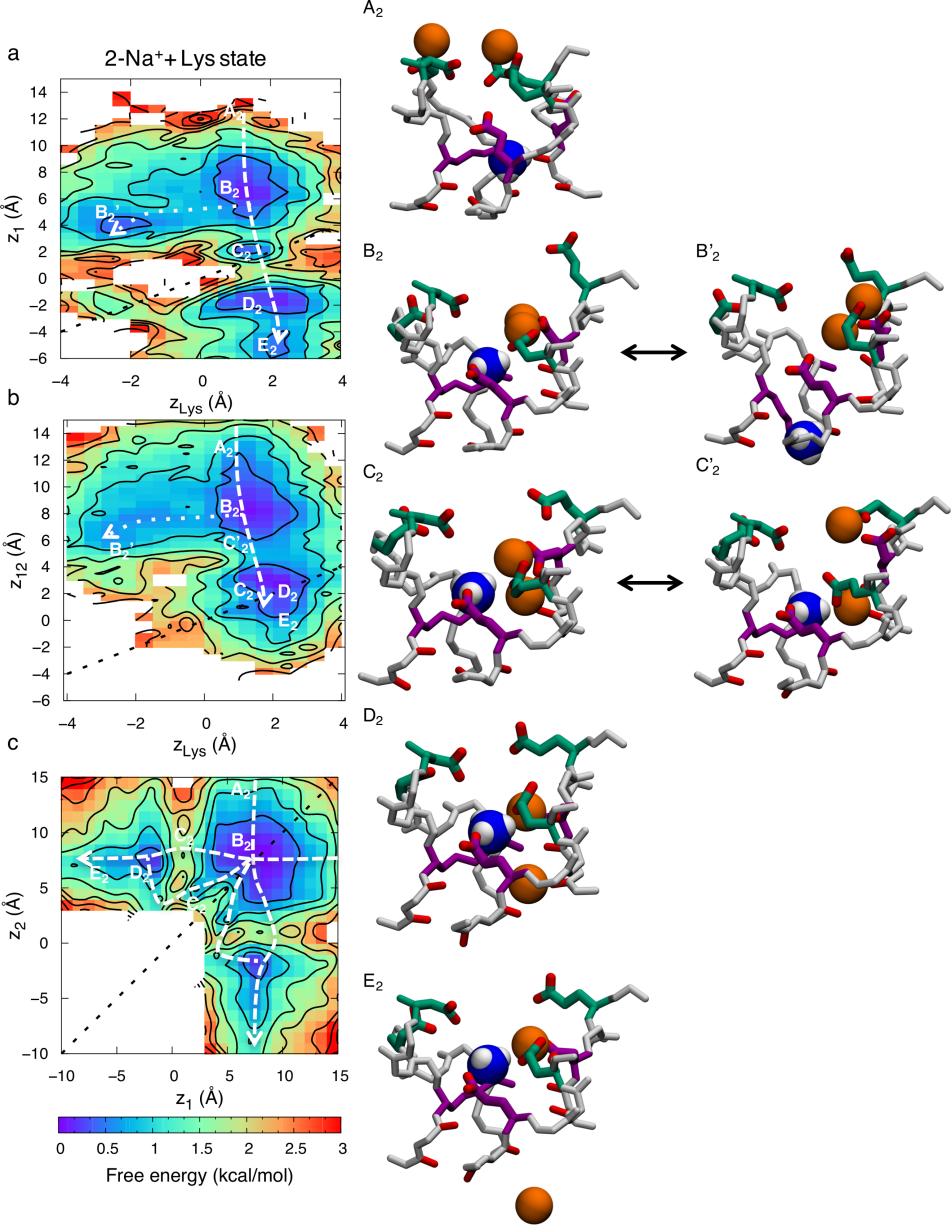
Two-ion permeation for Na^+^ in the Na_v_1.2 SF with protonated Lys^+^. 2D free energy projections showing the 2-ion conduction mechanism for: a) the bottom ion and Lys; b) the two ions and Lys; and c) the two ions; where *z*_1_ and *z*_2_ correspond to the *z* positions of the bottom and top ions, *z*_12_ the *z* position of the COM of the two ions, and *z*_Lys_ the *z* position of the Lys^+^ ammonium group. Snapshots, with the DEKA ring in purple, EEDD ring in green, and the charged ammonium group of the Lys shown as blue and white balls, indicate the Na^+^ ion (orange balls) movements.

The 2-ion conduction mechanism is summarized in Fig 7. The free energy projection in Fig 7A shows the position of the bottom ion (*z*_1_) as a function of the Lys (*z*_Lys_), Fig 7B shows the COM of the two ions (*z*_12_) as a function of the Lys, while Fig 7C shows bottom versus top ions. In this 2-ion case, we see five distinct states; state A_2_ is like the corresponding 1-ion state A_1_, but with an additional ion entering from the bulk above (*z*_12_~14 Å). In Fig 7B we can see how the two ions enter deeper into the SF and bind collectively to the inner and outer rings, just above the Lys in state B_2_ (*z*_1_~*z*_2_~7 Å & *z*_Lys_~2 Å). There is an additional state B_2_’, related to B_2_, where the ammonium group of the Lys is displaced downward (from *z*_1_~6 Å & *z*_Lys_~2 Å to *z*_1_~4 Å & *z*_Lys_~-2 Å; dotted line). However, just as for the 1-ion state, this downward Lys displacement is not sufficient for conduction, instead requiring that the ammonium group of Lys rise up and join one of the ions in a new intermediate stable state C_2_ as shown in Fig 7A (*z*_1_~*z*_Lys_~2 Å). This state only appears when there are 2 Na^+^ ions in the filter and seems to be vital for efficient permeation, because it allows the ion to pass the Lys with reduced energetic barrier (dashed line). The ion then binds below the Lys to the backbone carbonyls of the lower SF represented by state D_2_ (*z*_1_~-2 Å & *z*_Lys_~2 Å). In state E_2_ the top ion pushes the bottom ion into the cavity and binds to the inner ring (*z*_1_~-6 Å, *z*_2_~6 Å & *z*_Lys_~2 Å). Fig 7C shows only the two ions, at *z*_1_ (lower) and *z*_2_ (upper), and the corresponding states. The top ion can either enter and knock on or pass by the bottom ion. The dashed arrows show the lowest free energy path for conduction. The greatest barrier to overcome in the 2-ion state is 1.6±0.7 kcal/mol. Importantly, while conduction was seen to be possible with a singly-occupied SF (seen in Fig 6; dashed line), the entrance of a second Na^+^ ion enables a stable state (Fig 7A; state C_2_) where the ammonium group of the Lys and the bottom ion are collectively bound by the carboxylates, this allows the ion to pass by the Lys and in to the cavity with a lower barrier. As we shall see below, this efficient conduction mechanism does not exist for K^+^ ions.

### K^+^ exhibits less multi-ion complex formation, and is partially blocked by lysine

**Fig 8:**
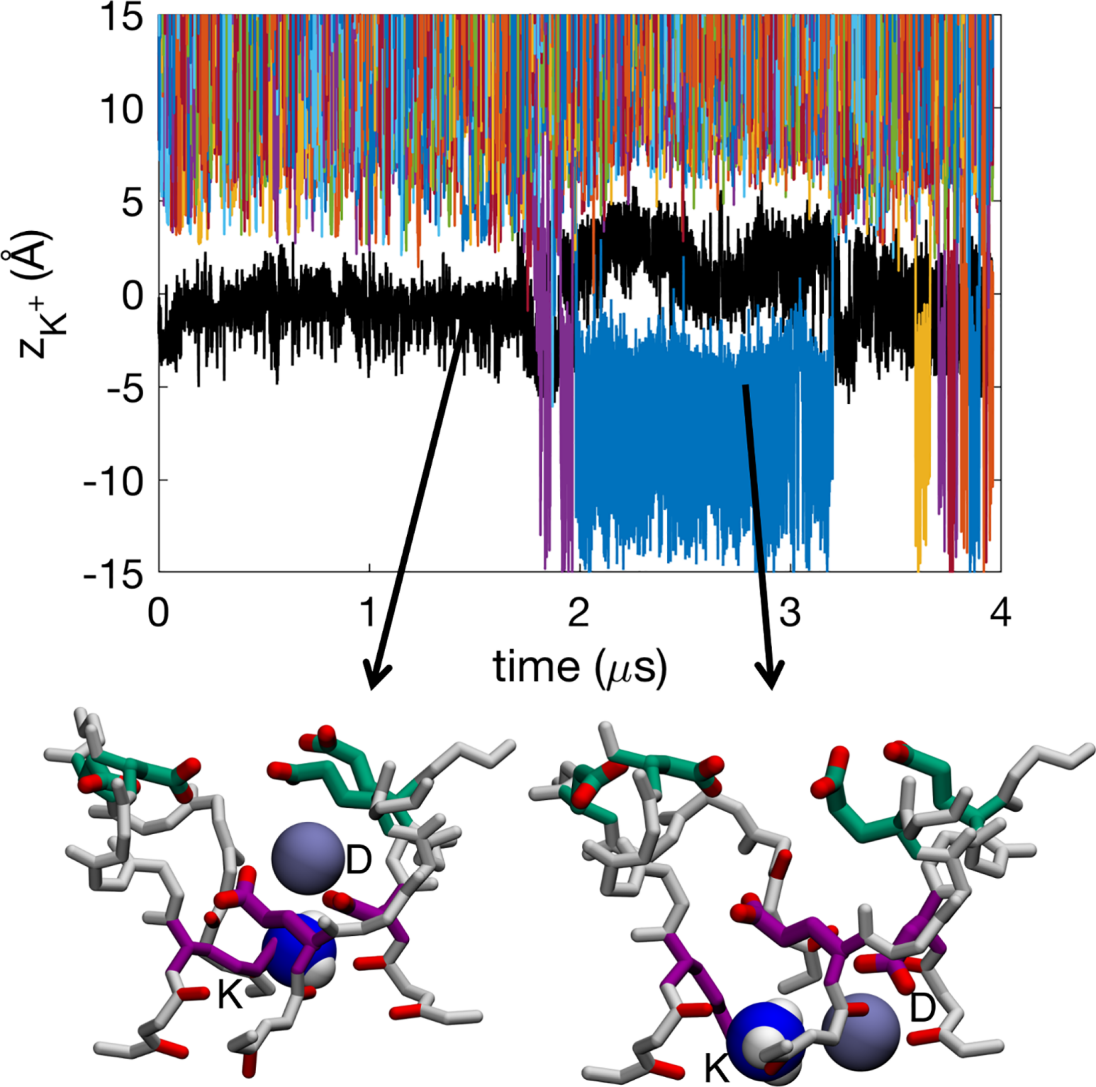
Time series of *z* positions for protonated Lys^+^ (K; black) and K^+^ ions (colors) in the SF of Na_v_1.2. Insets show snapshots of K^+^ ions (purple balls) in representative configurations with active residues from the DEKA (purple) and EEDD (green) rings labeled. The label for the Lys of DEKA, “K”, should not be confused with the potassium ion K^+^. The charged ammonium group of the Lys shown is shown as blue and white balls. See also sample trajectory in S3 Movie.

The Na_v_1.2 SF with charged Lys, in the presence of KCl solution, has an average occupancy of 1.3±0.1 K^+^ ions, being slightly lower than for Na^+^ ions, but comparable within errors. Fig 8 shows several ions (colored lines) entering and exiting the SF but not interacting extensively with the Lys (black line). We see 38 complete K^+^ ion permeation events (involving 13 distinct ions with repeat crossings, predominantly in the latter half of the 4 μs simulation after Lys rotamer change; see below and S3 Movie), being similar but somewhat less than the Na^+^ case above.

Most of the time there exists a single K^+^ ion in the SF (S9 Fig). When there are two K^+^ ions in the SF they are generally further from each other, with a mean ion-ion distance of 15.1±0.8 Å, compared to 11.4±1.2 Å for Na^+^. Snapshots in Fig 8 show representative configurations. The K^+^ ions most commonly bind to single carboxylates or stay unbound (S9 Fig). The lower occupancy and the larger ion-ion distances mean that we do not see the same tight multi-ion/multi-carboxylate complexes as frequently as we did for Na^+^ (S9 Fig), and when they do occur they are almost always singly coordinated by outer ring carboxylates (S1 Table; column 4). Instead, a single K^+^ ion typically enters the SF and binds solely to the D from the DEKA ring (S1 Table; column 6). The DEKA ring is involved in binding ~40% of the time, the EEDD ring ~70% of the time, and both rings cooperatively involved only ~10% of the time. This reduced binding and absence of complex formation deep in the SF slows K^+^ permeation. We instead observe K^+^ ions held bound to the D of DEKA, electrostatically repelled by the Lys ammonium group, until the Lys side chain changes rotamer downward to allow ion movement, in stark contrast to the Na^+^ case above.

**Fig 9:**
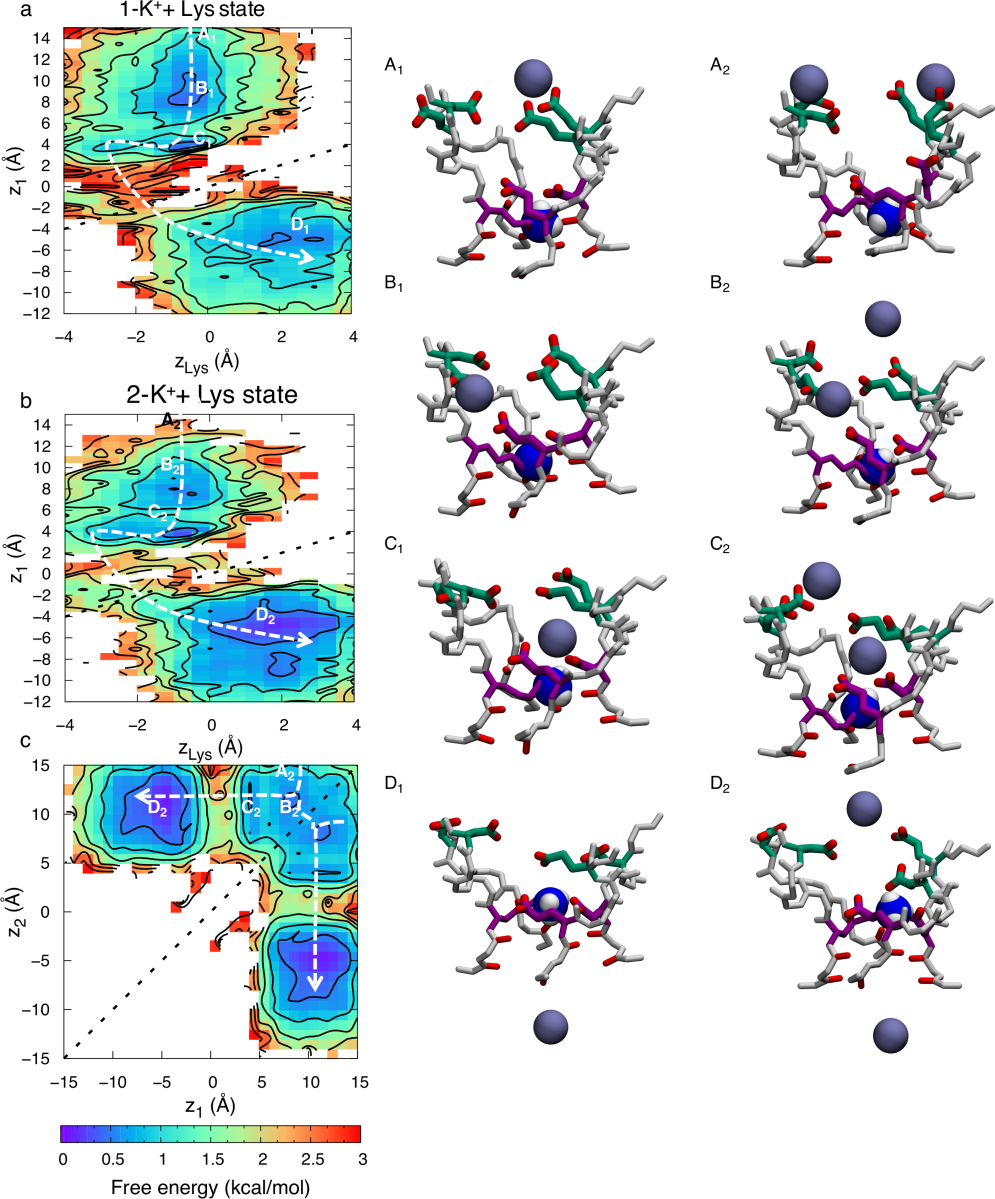
Permeation of K^+^ in the Na_v_1.2 SF with protonated Lys^+^. 2D free energy projections showing: a) 1- ion conduction mechanism; b & c) the 2-ion conduction mechanisms (graphed for the bottom ion and Lys in b, and the two ions and Lys in c); where *z*_1_ corresponds to the *z* position of the bottom ion, *z*_2_ the *z* position of the top ion, and *z*_Lys_ the *z* position of the Lys/K ammonium group. Snapshots for 1-ion (left) and 2-ion (right) mechanisms, with the DEKA ring in purple, EEDD ring in green, and the charged ammonium group of the Lys shown as blue and white balls, indicate K^+^ ion (purple balls) movements. State labels include a subscript 1 or 2, representing the 1-ion or 2-ion mechanisms, respectively.

We again see conduction with either 1 or 2 K^+^ ion occupancy. However, unlike for Na^+^ ions, we see similar permeation mechanisms when 1 or 2 K^+^ ions are in the SF. This, together with the large K^+^- K^+^ distance of 15.1±0.8 Å, compared to 11.4±1.2 Å for Na^+^, suggest that the second ion does not participate in conduction but rather is loosely associated around the vestibule region. Fig 9A shows the 1-ion conduction mechanism, with the ion at *z*_1_, and the ammonium group of Lys, at *z*_Lys_. We observe four states in the 2D PMF in Fig 9; A_1_, B_1,_ C_1_ and D_1_. In state A_1_ the ion is in the vestibular region of the SF (*z*_1_~14 Å) and the Lys is bound to one of the inner ring carboxylates (*z*_1_~0 Å). The ion then moves down and binds to the outer ring (*z*_1_~8 Å) represented by state B_1_. In state C_1_, the ion has entered deeper into the SF and is now bound only to the carboxylate of the D_1_ from the DEKA ring (*z*_1_~4 Å). The Lys is still bound to the inner ring in both these two states (*z*_K_~0 Å). The ions do not have considerable effect on the position of the ammonium group of the Lys. However, occasionally (~10% of the time) the Lys changes rotamer downward (*z*_Lys_<-2 Å), allowing leakage of K^+^ ions and thus permeation, represented by dashed line, leading to state D_1_. After the conduction event, the ammonium group of the Lys again bends upward to bind to the inner ring carboxylates (*z*_Lys_~2 Å). For this to happen the K^+^ ion has to pass by the ammonium group of the Lys in the lower part of the SF (*z*_1_~*z*_Lys_~-2 Å) where the electrostatic potential is less negative, leading to a larger energy barrier of 2.8±0.3 kcal/mol, as seen in Fig 8A along the pathway represented with a dashed arrow. This increased barrier (by 1.1±0.4 kcal/mol) would suggest a relative permeability for the channel of the order of 10, which is consistent with the experimental value for mammalian Na_v_ channels (e.g. P_Na+_/P_K+_ ~10 in Na_v_1.2 from rat [25]). This estimate, however, assumes the rate-limiting step for conduction does not involve Lys rotameric change, which was observed here on the multi-μs timeframe; apparently exceeding the sub-μs scale of permeation, but which may not have been reliably quantified based on 4μs simulation.

The 2-ion conduction mechanism in Fig 9B looks very similar, with the large K^+^- K^+^ distance of 15.1±0.8 Å showing that it is the same as the 1-ion conduction mechanism, only with an additional ion in the vicinity. Fig 9B shows the 2D PMF with the bottom ion, at *z*_1,_ and the ammonium group of Lys, at *z*_Lys_, being almost identical to the 1-ion case in Fig 9A; supporting the lack of a concerted 2-ion conduction mechanism. The lower ion enters the channel, binds collectively to the DEKA and EEDD rings in state B_2_, before binding solely to the DEKA ring in state C_2_. To enter the cavity, the ion has to wait for the Lys to change rotamer, it can then pass the Lys ammonium group in the lower SF, and then move further down into the cavity (state D_2_). In the meanwhile, the upper ion generally sits in the vicinity of the SF. In Fig 9C we see how the two ions can cross each other in the top part of the channel. The top ion can be anywhere between *z*_2_~7 Å and *z*_2_~15 Å during conduction, however, with a slight preference around *z*_2_~12 Å. The 2-ion energy barrier is similar to the 1-ion case at 2.7±0.3 kcal/mol. The Na^+^ and K^+^ barriers differ by 1.1 kcal/mol, however, the slowest coordinate in the K^+^ translocation pathway appears to involve the structural isomerization of the Lys side chain downward to permit conduction, leading to a permeation event that is more costly for K^+^.

Key to the low barrier for Na^+^ permeation is its ability to form tight multi-ion/multi-carboxylate clusters as well as a complex with Lys and 2 carboxylates, allowing pass-by conduction in the S_HFS_. We do not see the same tight K^+^-K^+^ clusters nor the simultaneous binding of K^+^ and Lys near the DEKA ring. Instead we see a lone K^+^ ion that is singly bound and forced to pass by the Lys lower in the SF (S9 Fig), where the electrostatic field is less favorable (Fig 3A), making the whole process far less likely.

We now explore the thermodynamic stabilities of the complexes observed during permeation events to understand the causes of the different Na^+^ and K^+^ mechanisms that may underlie selectivity.

### Multi-ion complex stability underpins selective conduction

Multi-ion complex formation with carboxylates is required for efficient permeation as it helps draw in and stabilize the ions in the SF. This acts as a precursor for the ion+Lys/multi-carboxylate complex that aids in the crossing of the SF. The analysis in S9 Fig suggests that such multi-ion/multi-carboxylate complexes appear more favorable for Na^+^ over K^+^. When the SF is occupied by a single ion, Na^+^ is most commonly bound to one or more carboxylate side chains (45% and 26%), whereas K^+^ is more likely to be unbound (61%) (S9B Fig), and Na^+^ is several times more likely than K^+^ to form multi-carboxylate complexes (26% vs. 6%). Of the multi-ion occupancies observed, Na^+^ ions are most likely to be either singly bound or in loose multi-carboxylate complexes (42% and 40%), whereas K^+^ is most likely to be either singly bound or unbound (52% and 36%). Na^+^ is several times more likely to form tight multi-ion/multi-carboxylate clusters than K^+^ (12% vs. 3%) (S9C Fig). For Na^+^ ions, these clusters are predominantly made up by outer ring carboxylates together with either the E or D from the DEKA ring, most commonly E_II_ together with D (S1 Table; column 3). For K^+^ such complexes are almost always made up only by the outer ring carboxylates (S1 Table; column 4).

**Fig 10:**
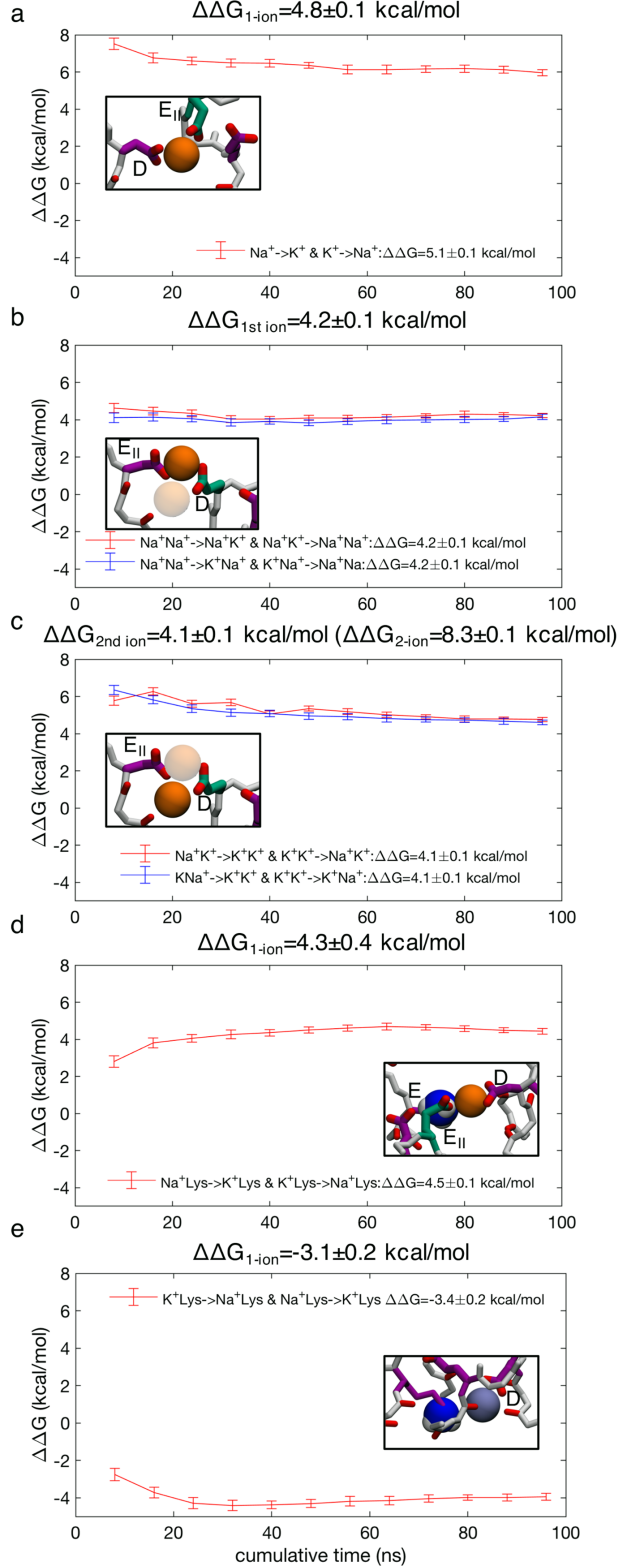
Free energy perturbation for ion binding selectivity. a) Single ion Na^+^→ K^+^ and K^+^→ Na^+^ in an ion-DE_II_ complex; b) 1^st^ ion transformations Na^+^Na^+^→K^+^Na^+^, Na^+^Na^+^→ Na^+^K^+^, K^+^K^+^→K^+^Na^+^ and K^+^K^+^→ Na^+^K^+^ in the 2-ion-DE_II_ complex (with 2^nd^ ion grayed out) and c) 2^nd^ ion transformations K^+^Na^+^→K^+^K^+^, Na^+^K^+^→ K^+^K^+^, K^+^Na^+^→Na^+^Na^+^ and Na^+^K^+^→ Na^+^Na^+^ (bottom) in the 2-ion-DE_II_ complex (with 1^st^ ion grayed out). d) single ion transformations Na^+^→K^+^ and K^+^→Na^+^ for the ion+Lys-DEE_II_ complex; e) single ion transformations K^+^→ Na^+^ and Na^+^→ K^+^ in an ion+Lys position in the lower SF (note that K^+^+Lys is the starting configuration in this case, leading to a negative free energy corresponding to the transformation K^+^→ Na^+^). The value for the bulk reference for Na^+^→K^+^ in aqueous solution was found to be 17.7 ± 0.3 kcal/mol. Insets show starting configurations and indicate amino acids involved. Free energy corrections to account for constraints used to maintain complexes (see text) were found to be: a) 0.30±0.02 kcal/mol; b) −0.017±0.042 kcal/mol; c) −0.023±0.075 kcal/mol; d) 0.15±0.42 kcal/mol; and e) 0.34±0.12 kcal/mol. Uncorrected ΔΔG values are displayed within each panel after the specified transformations, with corrected ΔΔG values given at the top of each panel, corresponding to 1-ion, or 1^st^ or 2^nd^ ion of a 2-ion complex (with the cumulative 2-ion value indicated in parenthesis in panel c). Snapshots, with the DEKA ring in purple, EEDD ring in green, and the charged ammonium group of the Lys shown as blue and white balls, indicate starting configurations with Na^+^ (orange balls) and K^+^ (purple balls).

Thus, tight 2-ion/multi-carboxylate complexes, similar to the complex that is formed before the low free energy pass-by conduction events for Na^+^ (Fig 7; state B_2_), are more common for Na^+^ than K^+^, and may be very important for selectivity (S9 Fig). FEP calculations were used to investigate the relative stabilities of Na^+^ and K^+^ in such a complex. The most representative multi-ion/multi-carboxylate complex was identified to be bound collectively by D and E_II_ (Fig 10B and 10C; inset) (69%) (S1 Table; column 3). Results from FEP calculations show preferential binding by Na^+^ with 4.8±0.1 kcal/mol for single ion occupancy (Fig 10A), showing a strong inherent preference by carboxylates to bind Na^+^, in agreement with Eisenman high field strength theory on ion selectivity [78]. Quantum mechanical calculations using a model DEKA ring have demonstrated a similar preference for Na^+^ over K^+^ (4.8 kcal/mol [79]), revealing consistency between models. The same complex containing two ions shows a large preference of 8.3±0.1 kcal/mol for Na^+^ over K^+^ ions (Fig 10B and 10C); 4.2 kcal/mol from the transformation of the 1^st^ ion (Fig 10B) and 4.1 kcal/mol from the transformation the 2^nd^ ion (Fig 10C). This shows that an additional ion creates extra stability for Na^+^ relative to K^+^. When another ion is added to the complex, the cumulative ion-carboxylate attraction increases faster than the carboxylate-carboxylate and ion-ion repulsion, more so for Na^+^ than K^+^, giving additional stability to Na^+^ complexes. These complexes create deeper binding in the SF that aids crossing the ammonium group of the Lys. We note that this large cumulative free energy difference between Na^+^ and K^+^ ions to form a 2-ion complex with multiple carboxylates is not the key energy controlling the different permeation mechanisms in Figs 7 and 9, but represents the relative stability of a complex that is particular to Na^+^, and largely unseen for the K^+^ ion because of its low stability, helping explain the distinct mechanisms.

During the ion crossing of the charged Lys, the ion creates a stable transition state together with the Lys and multiple carboxylates (Fig 10D; inset). This ion-Lys state is bound collectively by the D and E from the DEKA ring, as well as E_II_ from the outer ring, and shows preferential binding of a single Na^+^ by 4.3±0.4 kcal/mol (Fig 10D). This ability to create a joint ion-Lys complex to facilitate crossing of the Lys in the S_HFS_ is the salient difference in Na^+^ and K^+^ permeation mechanisms_._

The K^+^ ion instead usually resides alone in the SF and crosses the ammonium group of the Lys further down, (Fig 10E; inset), where the electrostatic fields arising from carboxylates in the upper SF is less attractive. If we examine the relative free energies of Na^+^ and K^+^ ions in this region of the SF near the Lys, where such crossing occurs for K^+^, we calculate a similar but reduced value of 3.1±0.2 kcal/mol, still favoring Na^+^ (Fig 10E). This value remains negative because the ion remains in contact with D and E side chains from the DEKA ring as it passes the Lys side chain. Theoretically, both K^+^ and Na^+^ should be able to translocate in this fashion when the Lys moves downward, but this was not observed for Na^+^. We may understand this from the relative destabilization of Na^+^ in this region relative to the selective multi-carboxylate complexes that form above it, making downward movement of Na^+^ less likely.

We therefore hypothesize that selective permeation in Na_v_1.2 arises from the distinct mechanisms (Lys-complex pass-by Na^+^ permeation versus electrostatically Lys plugged K^+^ permeation), which originates from the difference in stabilities of multi-ion and ion-Lys complexes in the high field strength region of the SF. Multi-ion/multi-carboxylate complexes have also been seen to be crucial for Na^+^ permeation in the bacterial Na_v_Ab channel, where a preference for single Na^+^ ions of -2.2±0.6 kcal/mol has been estimated [30]. In the bacterial channel, we also suggest that optimal conduction requires the greater occupancy of the 3-ion state which is achieved when the crystallographic E177-S178 H-bond is lost on 2 opposite monomers, allowing the S_HFS_ carboxylate groups to reach toward the center of the SF and coordinate 2 Na^+^ ions concurrently. This two-Na^+^/two-carboxylate complex offers additional stability, allowing those 2 ions to coexist in S_HFS_, and we calculate the relative stability of Na^+^ vs. K^+^ in S_HFS_ to become -3.8±0.2 kcal/mol in Na_v_Ab. This observation suggests that Na^+^ ions adopt a specific 3-ion arrangement with 2 ions at the level of S_HFS_, that does not occur in simulations of K^+^, which closely parallels our observations in the Na_v_1.2 channel.

### Conclusions

The ability to select for native ions while discriminating against other similar ions, is one of the key features of voltage gated ion channels. Ion conduction is a dynamic process that involves conformational isomerizations of the residues of the SF, as well as cooperation between multiple ions, requiring long timescale simulations to extract the underlying energetic landscape. In the absence of a high-resolution structure of a mammalian channel to study selectivity and conduction at the molecular level, we designed a model of the human Na_v_1.2 by patching the essential sequence of residues in and around the SF, including the key DEKA and EEDD rings, into the Na_v_Rh bacterial channel. This Na_v_Rh/Na_v_1.2 channel relaxes into an asymmetrical configuration, where the DEKA and EEDD rings are highly flexible and cooperate to ensure efficient conduction and selectivity for Na^+^ over K^+^, in line with previous studies of bacterial Na_v_ channels where similar flexibility has been shown to be critical for ion conduction [29, 30].

The presence of a positively charged Lys in Na_v_ channels has long been a source of intrigue; being essential for selectivity, but its specific role unexplained. Previous computational studies of eukaryotic Na_v_ models point toward a passive, hindering role, where the Lys side chain needs to bind away from the middle of the pore lumen in order to allow conduction of Na^+^ ions. We reveal a more intimate role for this residue, depending on its protonation state. When Lys is deprotonated (neutral), we observe a 2 to 3-ion occupancy in the SF where both 2- and 3-ion knock on conductions are common, facilitated by tight multi-ion/multi-carboxylate complexes that are made possible by the flexible side chains of the SF. When Lys is instead protonated (charged), we see reduction to 1 to 2-ion occupancy, due to the extra charge introduced by the ammonium group of the Lys. In fact, we see the Lys actively participating in the conduction mechanism with a role similar to that of a permeating ion. In the presence of K^+^ ions, the conduction mechanism instead relies on single ion permeation, where multi-ion/multi-carboxylate complexes are less likely.

These multi-Na^+^/multi-carboxylate complexes help attract Na^+^ in to the SF of Na_v_1.2, where binding deeper in the SF stabilizes the ions. Interestingly, the carboxylates involved in these key multi-Na^+^/multi-carboxylate complexes generally include only one carboxylate from DEKA and one or two from the EEDD ring in the vestibular region, rather than both D and E from the DEKA ring. This observation is consistent with mutagenesis experiments showing the need for only one of the D and E from the DEKA ring, supporting the idea of involvement of the outer ring [19]. These Na^+^ favorable complexes are more commonly bound collectively by D and E_II_. Cysteine mutations of the residues in the EEDD ring show the greatest decrease in conduction when this domain II Glu (E_II_ in our model) is mutated [25, 41], consistent with our observation that this residue from the outer ring is particularly important.

In the case of charged Lys we see conduction that is possible with a singly occupied SF, however, the binding of a second Na^+^ ion reduces the conduction barrier by creating an intermediate state that enables efficient multi-ion conduction. The second Na^+^ ion helps to push the ion already in the SF and stabilize a state where the ammonium group of the Lys and the bottom ion are collectively bound by the carboxylates, allowing the ion to pass by the Lys into the cavity. The facilitating of inward conduction by the presence of an additional external Na^+^ ion may explain the robust inward rectification of current in mammalian Na_v_ channels seen experimentally [1]. Even though more space for conduction can be created in the SF by the steric unplugging of the ammonium group of the Lys, no conduction occurs in that state. This behavior is consistent and comparable with previous results in Na_v_Ab, where pass by of ion by another would only occur at the level of S_HFS_. When the charged ammonium group of Lys is in the S_HFS,_ where the electrostatic potential is most negative, it creates a smooth electrostatic environment leading into the cavity, whereas when it is in the down state it creates a zone of high electrostatic potential that cuts the cavity off from the SF. The Na^+^ ion can benefit from the smooth energetic surface by binding at S_HFS_ in a multi-ion complex with Lys, whereas K^+^ cannot; instead becoming partially electrostatically blocked by the Lys in the lower SF. This illustrates the fundamental role of the Lys as an active participant in the selective conduction mechanism. K^+^ is far less likely to form these multi-ion/multi-carboxylate complexes; in particular, we see preferential binding of 8.3±0.1 kcal/mol for Na^+^-Na^+^ over K^+^-K^+^ in these complexes. Furthermore, K^+^ does not form the stable multi-carboxylate/Lys state. In fact, the ion+Lys/multi-carboxylate cluster favors Na^+^ over K^+^ by 4.3±0.4 kcal/mol. The consequence of this difference in affinity is a completely different conduction mechanism for K^+^, having to pass the ammonium group of Lys in the lower part of the SF, where the electrostatic environment is not as favorable. Interestingly, the apparently independent conduction pathways for Na^+^ and K^+^ ions could potentially explain absence of anomalous mole fraction effects in mammalian Na_v_ channels [53]. In the future, simulations of a model human Na_v_ channel in an open conformation, based on constrained-open Na_v_Ab or Na_v_Ms structures [9, 16, 27], could be used to explore the competition of Na^+^ and K^+^ ions under the driving force of a membrane potential.

It has been proposed that mammalian Na_v_s select for Na^+^ via a different mechanism to bacterial Na_v_s, owing to their divergent S_HFS_ site forming residues (EEEE Vs DEKA) [18, 80]. While this appears to be the case based on our observations, we do see evidence for common features that may be central to Na^+^ selectivity. Both bacterial and human channels make use of an efficient multi-ion process enabled by flexible carboxylates binding two Na^+^ ions during ion knock on or pass by conduction events. The efficiency of this multi-ion mechanism is reliant on the formation of 2-Na^+^/2-carboxylate complexes, whose thermodynamic stabilities are increased for Na^+^ compared to K^+^. Even though bacterial channels possess 4 carboxylates at the S_HFS_ site, only two are needed for high affinity binding [30]. Thus, the bacterial channel uses two of its four EEEE Glus, whereas the human channel uses carboxylates both from the DEKA and EEDD rings, leading to a Na^+^ permeation process that closely resembles our observations in the model human channel. However, the Lys residue in the human DEKA signature participates in the multi-ion knock-on mechanism in ways that impose a unique ion discrimination process, perhaps explaining the higher level of ion selectivity in mammalian Na_v_s [1]. We propose that this additional selectivity is achieved by establishing distinct permeation mechanisms for Na^+^ and K^+^ ions, where the Lys expedites Na^+^ translocation through high field strength complex formation, while partially blocking the K^+^ ion.

## Supporting information

S1 Text Supplementary methods for *Selective ion permeation involves complexation with carboxylates and lysine in a model human sodium channel*. (PDF)

S1 Table Summary of ion binding in the Na_v_1.2 SF of the Na_v_Rh/Na_v_1.2 channel. The first column defines which carboxylate(s) the ion(s) are bound to. Columns 2,3 and 4 show the tight 2-ion/multi-carboxylate clusters for the Na_**v**_1.2 SF with deprotonated Lys^**0**^ in NaCl, protonated Lys^**+**^ in NaCl, and protonated Lys^**+**^ in KCl solutions, respectively. Columns 5 and 6 show the tight ion+Lys/multi-carboxylate clusters with protonated Lys^**+**^ in NaCl and KCl solutions, respectively. Clusters that occur less than 4% of the time are not shown. Most commonly, Na^**+**^ is bound to D and/or E together with E_**II**_, whereas K^**+**^ is bound to the outer EEDD ring or singly bound to only the DEKA D side chain.
(TIFF)

S1 Fig Structural alignments of Na_v_ channels. Alignments of model human Na_**v**_1.2 (pink) cockroach Na_**v**_PaS (purple) and eel Na_**v**_1.4 (blue), showing DI and DIII on top and DII and DIV at the bottom. Panel a) shows the alignment according to the backbone of the SF and outer vestibule based on all four subunits, while b) shows alignment according to the SF and vestibule subunit by subunit, revealing the similarity of the Na_**v**_1.2 model to Na_**v**_PaS and EeNa_**v**_1.4 cryoEM structures. Side chains of Glu, Asp and Lys are indicated with sticks.
(TIF)

S2 Fig Na^+^ ions in the SF of bacterial Na_v_Ab. 2D free energy projections showing: a) 2-ion occupancy; and b & c) 3-ion occupancy (graphed for all three ions in b, and the top two ions in c); where *z*_**1**_, *z*_**2**_, and *z*_**3**_ correspond to the *z* positions of the bottom, middle and top ions, respectively, and where *z*_**23**_ is the *z* position of the COM of the top two ions. When there are 2 ions in the SF, the ions are trapped and it is not until a third ion enters from above that we can observe either a knock on or pass by conduction. Snapshots, with the EEEE ring in purple, indicate the corresponding Na^**+**^ ion (orange balls) movements. State labels include a subscript 2 or 3, representing the 2-ion or 3-ion mechanisms, respectively. Here the state label A has no subscript because A_**2**_ and A_**3**_ represent the same state, with same 2-ion configuration in the SF, but with approaching 3^**rd**^ ion from bulk.
(TIF)

S3 Fig Radial distribution functions. Distributions are shown for Na^**+**^-Na^**+**^ (a), K^**+**^-K^**+**^ (b), Na^**+**^-carboxylate (c) and K^**+**^-carboxylate (d). Multi-ion clusters defined by ***r*** < **4.7** Å for Na^**+**^ and ***r*** < **5.5** Å for K^**+**^, and carboxylate binding by ***r*** < **3.8** Å for Na^**+**^ and ***r*** < **4.2** Å for K^**+**^.
(TIF)

S4 Fig RMSD for the grafted region of the channel (residue numbers 174–184). a) Na^**+**^ with deprotonated Lys^**0**^; b) Na^**+**^ with protonated Lys^**+**^; and c) K^**+**^ with protonated Lys^**+**^. Red dashed lines indicate the level of RMSD after the equilibration period. Late changes due to the onset of conduction, particularly for K^**+**^, are discussed in the text.
(TIF)

S5 Fig Time series of C_α_ vertical positions for Na_v_1.2 DEKA and EEDD residues. a) Na^+^ with deprotonated Lys^0^; b) Na^+^ with protonated Lys^+^; and c) K^+^ with protonated Lys^+^.
(TIF)

S6 Fig Mean backbone fluctuations. Fluctuations are shown for residues 178 to 184 in Na_**v**_Rh/Na_**v**_1.2 (yellow) and Na_**v**_Ab (blue) for: a) the mean of the four subunits (where orientation was according to all 4 subunits); and b) each individual subunit (where orientation was according to the subunit of interest).
(TIF)

S7 Fig Ion coordination inside the channel. Mean ion coordination numbers relative to bulk water in Na_**v**_Rh/Na_**v**_1.2 for: a) Na^**+**^ with deprotonated Lys^**0**^; b) Na^**+**^ with protonated Lys^**+**^; and c) K^**+**^ with protonated Lys^**+**^. Bulk hydration numbers are 5.67±0.04 for Na^**+**^, and 6.94±0.07 for K^**+**^.
(TIF)

S8 Fig Occupancy analysis in the channel with neutral Lys^0^ (deprotonated K of DEKA). a) Distribution of Na^+^ occupancy and dominant clusters representing complexes with: b) 1-ion occupancies; c) 2 ion occupancies; and d) 3 ion occupancies. In panels c and d, ‘single’ clusters include 1-ion/1-carboxylate complexes only, 'multi' clusters include 1-ion/multi-carboxylate complexes with a loosely associated second ion, and ‘tight multi’ clusters represent 2-ion/multi-carboxylate clusters where both ions are within an ion-ion cut off, defined from the radial distribution function *g*(*r*) for ion-ion distance (see text). The channel range is defined by the region -15<*z*<15 Å.
(TIF)

S9 Fig Ion occupancies for the Na_v_Rh/Na_v_1.2 SF with charged Lys^+^ (protonated K or DEKA). a) Distribution of ion occupancies. Panels b and c show dominant cluster conformations for: 1-ion (b) and 2 ion (c) occupancies, for Na^+^ (orange) and K^+^ (blue). See S8 Fig for definitions.
(TIF)

S10 Fig Free energy convergence analysis in the Na_v_Rh/Na_v_1.2 channel for Na^+^ with deprotonated Lys^0^. Panel a shows the convergence of 1D free energy for the ion across the channel, comparing time ranges specified in the legend. The free energy profile converges quickly in the outer vestibule and SF, but more slowly in the central cavity because it takes longer for ions to enter this part of the channel. Panels b-e show convergence of 2D free energy maps corresponding to Fig 4, for the last 2 *μ*s of each simulation (left and right show time ranges 0–1 ***μ***s and 0–2 ***μ***s, respectively).
(TIF)

S11 Fig Free energy convergence analysis in the Na_v_Rh/Na_v_1.2 channel for Na^+^ with protonated Lys^+^. Panel a shows the convergence of 1D free energy for the ion across the channel, comparing time ranges specified in the legend. Panels b-e, show convergence of 2D maps corresponding to Figs 6 & 7 (left and right show time ranges 0–3 ***μ***s and 0–4 ***μ***s, respectively).
(TIF)

S12 Fig Free energy convergence analysis in the Na_v_Rh/Na_v_1.2 channel for K^+^ with protonated Lys^+^. Panel a shows the convergence of 1D free energy for the ion across the channel, comparing time ranges specified in the legend. Panels b-d show convergence of 2D maps, corresponding to Fig 9 (left and right show time ranges 0–3 ***μ***s and 0–4 ***μ***s, respectively).
(TIF)

S1 Movie Sodium ion movement in the Na_v_1.2 SF with neutral Lys^0^. Sample trajectory revealing interactions of Na^+^ ions with the Na_v_1.2 SF, showing multi-ion complexes involving carboxylates from inner DEKA and outer EEDD rings, and knock-on and pass-by conduction events. Na^+^ ions are shown as orange balls, lysine N atom as a blue ball, and side chains of DEKA and EEDD rings as purple and green sticks, respectively.
(MP4)

S2 Movie Sodium ion movement in the Na_v_1.2 SF with charged Lys^+^. Sample trajectory revealing interactions of Na^+^ ions with the Na_v_1.2 SF, showing multi-ion complexes involving inner DEKA and outer EEDD rings, and complex formation with the lysine ammonium group, enabling pass-by conduction events. Representations as in S1 Movie.
(MP4)

S3 Movie Potassium ion movement in the Na_v_1.2 SF with charged Lys^+^. Sample trajectory revealing interactions of K^+^ ions with the Na_v_1.2 SF, showing ion interactions with DEKA and EEDD rings, lack of multi-ion or lysine complex formation, and conduction events requiring unblocking of the SF by downward movement of the lysine ammonium group. Representations as in S1 Movie, with K^+^ ions shown as purple balls.
(MP4)
