# Supplementary material for: Selective ion permeation involves complexation with carboxylates and lysine in a model human sodium channel
Source: PLoS Comput Biol. 2018 Sep 12;14(9):e1006398. doi: 10.1371/journal.pcbi.1006398 (PMC6152994; doi:10.1371/journal.pcbi.1006398)
Supplement: S1 Text — (PDF) [file pcbi.1006398.s001.pdf]

Supplementary Information

for

Selective ion permeation involves complexation with carboxylates and lysine in a model human sodium channel.

Emelie Flood, Céline Boiteux and Toby W. Allen*

School of Science, RMIT University, Melbourne, Vic, 3001, Australia.

* Corresponding author: toby.allen@rmit.edu.au

Supplementary Methods

Model $Na_v1.2$ Channel Creation

A model of the human $Na_v1.2$ channel was created by using the bacterial Na_vRh as a scaffold, for reasons outline in the main text Methods section. The structures of the two channels were aligned (see Fig.1) and the residues in and around the SF mutated into Na_vRh residues. The mutated region contained the key DEKA-ring as well as the vestibular EEDD-ring and surrounding residues believed to be important (see Methods). Models were created both with charged and neutral DEKA Lys, and proteins were then relaxed using Molecular Dynamics (MD) simulation in hydrated lipid bilayers, as detailed in the following section. Additional procedures not described in the main text Methods section are discussed below.

Rebuilding missing intracellular loops: Prior to sequence patching, modifications to the Na_vRh structure were required, owing to the absence of intracellular loops connecting S2 and S3 helices in the VSD. The Na_vRh structure was completed by rebuilding the missing loops (residue 70-75, Na_vRh numbering) in VSD II using Rosetta [1], chosen as it is the VSD with most residues resolved in the crystal structure of Na_vRh. This loop was then grafted on to the other VSDs, based on the assumption that these loops will be similar in all VSDs [2], minimizing the number of residues being rebuilt. The loop conformation chosen was representative of the best cluster in Rosetta and favors the positioning of aromatic residues F72 and F73 at the lipid interface, while charged residues D71 and K74 reside in the aqueous environment. Torsional angles of the connecting parts of the model were energy minimized without changing the side chain or backbone conformation of the rest of the protein. During loop optimization, the original X-ray structure rotamer orientations were used for residues not being rebuilt to keep the model as close as possible to the initial structure.

Maintaining key hydrogen bonds: There is a hydrogen bond present in both Na_vRh and Na_vAb structures between Thr178 and Trp182 that has been postulated to be important for keeping the shape of the SF in bacterial Na_v [2,3]. These residues are conserved throughout the Na_v family and all but one is present in human $Na_v1.2$ (T382-W386, C940-W943, T1420-W1424, T1712-W1716; $Na_v1.2$ numbering). To make sure that the mutations do not disturb the shape of the SF, this bond was therefore constrained throughout the mutation, minimization and equilibration of our model, and then released for unbiased production simulations.

Molecular Dynamics Simulation Details

The proteins were embedded in lipid bilayers of dipalmitoyl-phosphatidylcholine (DPPC), being the best characterized lipid for MD simulations [4], with explicit TIP3P water molecules [5] and 150 mM of NaCl or KCl solution. Systems were built and pre-equilibrated with CHARMM [6] and further equilibrated using NAMD [7] using the CHARMM36 [4] lipid and CHARMM22 protein and ion parameters [8] with CMAP corrections [9], to be consistent with previous simulations of the bacterial Na_vAb [10].

A number of attempts to adjust the extent of ion pairing between Na^+ and K^+ interacting with Glu/Asp carboxylate oxygen atoms have been made in the past by adjusting Leonard-Jones (LJ) parameters with a non-bonded fix (NBFIX) according to hydration free energy and osmotic pressure data [11,12,13]. These investigations had an overall similar conclusions on ion selectivity when using modified and unmodified parameters for LeuT [11], a Na^+/Ca^{2+} exchanger [12], and an acid sensing ion channel [14]. Furthermore, previous simulations of ion pairing between Na^+ and acetate have shown reasonable agreement between standard LJ parameters and quantum mechanical *ab initio* MD Umbrella Sampling calculations, and suggest that addition of NBFIX adjustment makes the ion-carboxylate pairing far too repulsive, despite improving the description of osmotic pressure [14]. The binding affinities and dissociation constants (K_D) using either the unmodified Lorentz-Berthelot mixing rule or NBFIX modifications showed general agreement with experiment [14]. We have previously shown, as expected, that changing to a more repulsive NBFIX interaction between ions and carboxylates leads to a reduction in ion-carboxylate pairing, but that even then Na^+ maintains increased multi-ion complexes compared to K^+ [14]. Therefore, we have used standard parameters for the ion-carboxylate interaction in this study, and refer to Ref.[14] for more information on the effects of NBFIX on ion-carboxylate complex formation.

Adjustments to the LJ NBFIX for ions interacting with backbone carbonyl oxygen atoms (ϵ_0=−0.102 and r_{min}=3.64 Å for K^+-O and ϵ_0=−0.0750 and r_{min}=3.30 Å for Na^+ -O) have previously been made to yield free energies of ion solvation (of -80 kcal/mol and -101 kcal/mol for K^+ and Na^+) in the protein backbone mimetic, N-methyl-acetamide that match free energies of ion hydration [15]. These parameters have been previously used for narrow K^+ channels [16] and also adopted for these simulations to better describe Na^+ and K^+ interacting with backbone carbonyl oxygen atoms (during Anton production runs, described below, these were only applied to SF residues 178-181 due to a limitation in number of NBFIX modifications permitted).

During equilibration simulations using NAMD 2.9 [17], the NPT ensemble was imposed using a Langevin piston to maintain constant pressure of 1atm [18,19], and a Nosé-Hoover thermostat to maintain constant temperature of 323K [20,21]. The RATTLE algorithm [22] was used to maintain the bonds to hydrogen atoms. Electrostatic interactions were calculated for the tetragonal periodic box using Particle Mesh Ewald [23] with grid spacing of 1.5 Å and 6th order B-spline for mesh interpolation. The non-bonded pair list was built to 16 Å, with a real space cut off at 12 Å and force switching from 10 Å. The protein was constrained while the membrane was energy minimized for 1000 steps and then equilibrated by initially constraining all heavy atoms with a 10 kcal/mol/Å^2 harmonic restraint, which was then slowly released over 2.5 ns. Residue 182 in subunit II was removed and residue 181 and 183 pulled together in steps of 0.1 Å with a force constant of 100 kcal/mol/Å^2, decreased over 20 ns, while the other subunits and residues outside of the SF were constrained during this adjustment, with protein heavy atoms constrained using 10 kcal/mol/Å^2 harmonic constraints. Restraints with force constant 10 kcal/mol/Å^2 on the hydrogen bonds between T382-W386, C940-W943, T1420-W1424 and T1712-W1716 were added, and the protein was equilibrated for an additional 100 ns. Unbiased production simulations were then carried out on the purpose-built supercomputer Anton [24,25] for 4 μs for Na_vRh/Na_v1.2 with charged Lys both for NaCl and KCl, and 2 μs for neutral Lys (less time required due to better

sampling of ion movements), totaling 12 μs. The NPT ensemble was maintained on Anton with a Nose-Hoover thermostat [20,21] and a Martyna-Tobias-Klein barostat [26] at 323 K with a 2 fs time step. A cut-off of 12.72 Å was used and long-range electrostatic forces evaluated with Gaussian split Ewald method every 6 fs [27,28,29].

Simulation Analysis

To determine the number of ions involved in ion conduction, the occupancy in the pore was calculated based on the mean and standard deviation of the number of ions within $\mathrm{r} < 10$ Å and $-15 < \mathrm{z} - \mathrm{z}_{ref} < 15$Å, where $\mathrm{z}_{\mathrm{ref}}$ is the z position of the center of mass (COM) of the backbone atoms of residues 178-182 (incorporating the DEKA as well as two additional residues above and two residues below); chosen to encompass entire $\mathrm{Na_vRh/Na_v1.2}$ pore, including accessible region of the intracellular gate, central cavity, SF and the outer vestibule. This outer cut off of z=+15 Å is consistent with our analysis of the vestibular ring structure, such that this range always includes the EEDD side chains. Compared to previous studies with $\mathrm{Na_vAb}$ [10], the outer cut off was extended by 2.5 Å to allow for this broader binding region in the outer vestibule. Permeation events were counted every time an ion crossed the SF and passed the Lys (inward moving ion starting at $z > 5$ Å and reaching $z < z_{Lys}$ and $z < -2$ Å; reversed for an outward permeating ion).

The free energy map, or potential of mean force (PMF), for ion movement through the filter was calculated for each system using trajectory data with specific ion occupancies, to ensure well-defined free energy maps. The relevant occupancies for permeation were found to be either 2 or 3 ions for the channel with neutral Lys, and 1 or 2 ions for the channel with charged Lys. The PMFs were calculated from unbiased simulations as

$$W(\{z_i\}) = -k_B T \ln \rho(\{z_i\}) + C,$$

where ρ is the unbiased probability distribution as a function of reaction coordinate(s) $\{z_i\}$, being the vertical positions of one or more ions (z_1, z_2, or z_3; defined with z_1 being the lower-most ion), or their centroids (e.g. $z_{12} = (z_1 + z_2)/2$), each relative to z_{ref}, and where C is a constant. Errors for the maps were calculated by dividing the well-sampled part of the trajectory (the last 2 μs of the trajectory for NaCl and the last 2.2 μs trajectory for KCl) into two parts to determine the deviation between the maps. The total error for the maps was calculated as the root mean square, including all points with energy < 3 kcal/mol (to avoid poorly sampled regions at the periphery).

Structural changes, reported in S4 Fig, were investigated by root mean square deviation (RMSD) of the entire mutated region (residues 174-184). The RMSD of the mutated region was calculated to determine the stability of the model. S4 Fig shows how, although the model goes through initial structural changes, the RMSD becomes stable and changes subside. Experiments have shown that the SF of the mammalian $\mathrm{Na_v}$ is highly flexible [30], placing some limitation on direct structural comparisons between model and published eukaryotic structures. Therefore, the Root Mean Square Fluctuation (RMSF) was calculated for the backbone of each residue in the SF and vestibule (spanning only the SF residues 178-184) to evaluate the flexibility of the SF region only, reported in S6 Fig. The RMSF was measured after orientation with respect to the backbone of residues 178-184 for either all subunits together, or each

individual subunit, to investigate both relative and internal fluctuation, respectively. For comparative purposes, the same calculation was done for 2.5 μs long simulations with the bacterial channel Na_vAb. The $Na_vRh/Na_v1.2$ model was also compared structurally to the eukaryotic Na_vPaS and $EeNa_v1.4$ by performing RMSD calculations of the equilibrated $Na_vRh/Na_v1.2$ model to the cryo-EM structure of Na_vPaS and $EeNa_v1.4$, using the backbone of each residue in the SF and vestibule (residue 178-184). The stability of the H-bonds between T382-W386, C940-W943, T1420-W1424 and T1712-W1716 ($Na_v1.2$ numbering, corresponding to Thr178-Trp182 in Na_vRh) were evaluated by calculating the donor-acceptor distance over the course of the simulation.

To determine the important states involved in ion permeation, clustering analysis was performed. The frames were broken down according to ion occupancies and the representative occupations further analyzed. The radial distribution function for ion-ion and ion-carboxylate pair interactions was evaluated and a cutoff based on the first minimum was then determined (Na^+-Na^+:r_{min}=4.7 Å, K^+-K^+: r_{min} =5.5 Å, Na^+-carboxylate:r_{min}=3.8 Å, K^+- carboxylate:r_{min} =4.2 Å) (S3 Fig). This cutoff was used to assign configurations to clusters based on the number of DEKA and EEDD carboxylates within the ion-carboxylate cutoff and, in the case of multi-ion occupancies, whether or not the ions were within the ion-ion cutoff distance. The clusters were further broken down according to which residues the ions were bound to. The most stable frame of the most representative clusters was identified by choosing the configuration with highest probability of occurrence within each cluster.

The most important states involved in the permeation of Na^+ and K^+ ions across the SF were identified from the PMFs (Fig.3&4) and cluster analysis (S1 Table). These states include complexes involving one or more carboxylate groups, Na^+ or K^+ ions and/or the signature Lys ammonium group. To evaluate the relative binding affinities of these states, Free Energy Perturbation (FEP) [31] calculations were performed. The starting frame was chosen as the most stable frame of the cluster, as described above. The relative binding affinity, $\Delta\Delta G_{Na^+\to K^+}$, was calculated from

$$\begin{aligned}\Delta\Delta G_{Na^+\to K^+} &= \Delta G_{K^+:Bulk\to Site} - \Delta G_{Na^+:\,Bulk\to Site}\\ &= \Delta G_{Na^+\to K^+:Site} - \Delta G_{Na^+\to K^+:\,Bulk}\end{aligned}$$

The NAMD FEP module, using dual topology, was used to determine $\Delta G_{Na^+\to K^+:Site}$ and $\Delta G_{Na^+\to K^+:Bulk}$ by alchemically transforming Na^+ to K^+ and K^+ to Na^+ using a coupling parameter, λ. The coupling parameter was divided into 20 windows that were initiated by consecutively increasing λ in steps of 0.05 for 0.2 ns each. Each window was then equilibrated and simulated concurrently for 2.4 ns giving a total of 48 ns simulation per calculation. The windows were combined and the free energy difference calculated with WHAM [32]. Both forward and backward transformations were performed ($Na^+\to K^+$ and $K^+\to Na^+$), and for the case of a site bound by 2 ions, each individual ion was transformed separately and the final transformation calculated by the mean of the two transformations (1st ion: mean of $Na^+Na^+\to K^+Na^+$ and $Na^+Na^+\to Na^+K^+$, or mean of $K^+K^+\to K^+Na^+$ and $K^+K^+\to Na^+K^+$; 2nd ion: mean of $K^+Na^+\to K^+K^+$ and $Na^+K^+\to K^+K^+$; or mean of $K^+Na^+\to Na^+Na^+$ and $Na^+K^+\to Na^+Na^+$). The endpoint histograms for the adjacent λ for the forward and backward transformations were combined with the VMD plugin ParseFEP [33] using Bennett Acceptance Ratio (BAR) [34].

During these FEP calculations, ions were constrained with a 20 kcal/mol/Å^2 half-harmonic flat-bottomed constraint with outer radius of 2.75 Å from the COM of the multiple carboxylate carbon atoms for the ion/ion+Lys-carboxylate clusters. The results were adjusted for the constraint force by subtracting the Boltzmann average of the difference in constraint energy for $\lambda = 0$ and $\lambda = 1$. Most of the time the constraints were not interfering and thus only a small correction (no greater than 0.3 kcal/mol; computed as the difference in $-k_B T ln \langle \exp{(-\Delta U / k_B T)} \rangle_{(U_0(\lambda))}$between $\lambda = 0$ and 1, where $U_0(\lambda)$ is the unperturbed system potential and ΔU is the constraint energy) was subtracted from the results. For the case of ion+Lys in the lower SF, a constraint was applied to the ion and the ammonium group of the Lys, keeping them at that location (-4.0<z<-2.5 Å), using a flat-bottomed harmonic constraint with force constant 20 kcal/mol/Å^2. The correction for the constraint was found to be 0.34±0.12 kcal/mol. During the $Na^+ \rightarrow K^+$ and $K^+ \rightarrow Na^+$ transformations, a harmonic constraint of 100 kcal/mol/Å^2 was applied between the vanishing and appearing ions to maintain overlap. A convergence criterion of $\Delta\Delta G$ within 1 kcal/mol was used. The first 1.2 ns of each window was discarded for the single-ion/multi-carboxylate complex. For the 2-ion complex, the first 0.6 ns for the 1st and 1.4 ns for the 2nd ion transformation were removed from each window. For the ion+Lys complex, the first 1.0 ns was discarded, while for the ion adjacent to Lys in the lower SF the first 1.2 ns was discarded for each window.

To examine the electrostatic influence of SF signature sequences and vestibular charge rings on permeation, Poisson's equation was solved for long ranged electrostatic interactions using a smooth particle mesh Ewald method, using VMD plugin PMEPot [35], yielding electrostatic potential maps for the Na_vRh/Na_v1.2 and Na_vAb channels. A grid size of 1 Å was used and the final map in the SF was averaged over every 100th frame. The ion density was evaluated with the VMD VolMap plugin [36].

Supplementary References

1. Combs SA, DeLuca SL, DeLuca SH, Lemmon GH, Nannemann DP, et al. (2013) Small-molecule ligand docking into comparative models with Rosetta. Nature protocols 8: 1277-1298.
2. Payandeh J, Scheuer T, Zheng N, Catterall WA (2011) The crystal structure of a voltage-gated sodium channel. Nature 475: 353-358.
3. Zhang X, Ren W, DeCaen P, Yan C, Tao X, et al. (2012) Crystal structure of an orthologue of the NaChBac voltage-gated sodium channel. Nature 486: 130-134.
4. Klauda JB, Venable RM, Freites JA, O'Connor JW, Tobias DJ, et al. (2010) Update of the CHARMM all-atom additive force field for lipids: validation on six lipid types. The journal of physical chemistry B 114: 7830-7843.
5. Jorgensen WL, Chandrasekhar J, Madura JD, Impey RW, Klein ML (1983) Comparison of simple potential functions for simulating liquid water. The Journal of chemical physics 79: 926-935.
6. Brooks BR, Brooks CL, MacKerell AD, Nilsson L, Petrella RJ, et al. (2009) CHARMM: the biomolecular simulation program. Journal of computational chemistry 30: 1545-1614.
7. MacKerell Jr AD, Bashford D, Bellott M, Dunbrack Jr RL, Evanseck JD, et al. (1998) All-atom empirical potential for molecular modeling and dynamics studies of proteins†. The journal of physical chemistry B 102: 3586-3616.
8. MacKerell Jr AD, Bashford D, Bellott M, Dunbrack Jr RL, Evanseck JD, et al. (1998) All-atom empirical potential for molecular modeling and dynamics studies of proteins. The journal of physical chemistry B 102: 3586-3616.
9. MacKerell AD, Feig M, Brooks CL (2004) Extending the treatment of backbone energetics in protein force fields: Limitations of gas‐phase quantum mechanics in reproducing protein conformational distributions in molecular dynamics simulations. Journal of computational chemistry 25: 1400-1415.
10. Boiteux C, Vorobyov I, Allen TW (2014) Ion conduction and conformational flexibility of a bacterial voltage-gated sodium channel. Proceedings of the National Academy of Sciences 111: 3454-3459.
11. Noskov SY, Roux B (2008) Control of ion selectivity in LeuT: two Na+ binding sites with two different mechanisms. Journal of molecular biology 377: 804-818.
12. Marinelli F, Almagor L, Hiller R, Giladi M, Khananshvili D, et al. (2014) Sodium recognition by the Na+/Ca2+ exchanger in the outward-facing conformation. Proceedings of the National Academy of Sciences 111: E5354-E5362.
13. Venable RM, Luo Y, Gawrisch K, Roux B, Pastor RW (2013) Simulations of anionic lipid membranes: Development of interaction-specific ion parameters and validation using NMR data. The Journal of Physical Chemistry B 117: 10183-10192.

14. Lynagh T, Flood E, Boiteux C, Wulf M, Komnatnyy VV, et al. (2017) A selectivity filter at the intracellular end of the acid-sensing ion channel pore. eLife 6: e24630.
15. Noskov SY, Berneche S, Roux B (2004) Control of ion selectivity in potassium channels by electrostatic and dynamic properties of carbonyl ligands. Nature 431: 830-834.
16. Bernèche S, Roux B (2005) A gate in the selectivity filter of potassium channels. Structure 13: 591-600.
17. Phillips JC, Braun R, Wang W, Gumbart J, Tajkhorshid E, et al. (2005) Scalable molecular dynamics with NAMD. Journal of computational chemistry 26: 1781-1802.
18. Andersen HC (1980) Molecular dynamics simulations at constant pressure and/or temperature. The Journal of chemical physics 72: 2384-2393.
19. Feller SE, Zhang Y, Pastor RW, Brooks BR (1995) Constant pressure molecular dynamics simulation: the Langevin piston method. The Journal of chemical physics 103: 4613-4621.
20. Nosé S (1984) A unified formulation of the constant temperature molecular dynamics methods. The Journal of chemical physics 81: 511-519.
21. Hoover WG (1985) Canonical dynamics: equilibrium phase-space distributions. Physical review A 31: 1695.
22. Andersen HC (1983) Rattle: A "velocity" version of the shake algorithm for molecular dynamics calculations. Journal of Computational Physics 52: 24-34.
23. Darden T, York D, Pedersen L (1993) Particle mesh Ewald: An N-log(N) method for Ewald sums in large systems. J Chem Phys 98: 10089.
24. Shaw DE, Dror RO, Salmon JK, Grossman J, Mackenzie KM, et al. Millisecond-scale molecular dynamics simulations on Anton; 2009. IEEE. pp. 1-11.
25. Shaw DE, Grossman J, Bank JA, Batson B, Butts JA, et al. Anton 2: raising the bar for performance and programmability in a special-purpose molecular dynamics supercomputer; 2014. IEEE Press. pp. 41-53.
26. Martyna GJ, Tobias DJ, Klein ML (1994) Constant pressure molecular dynamics algorithms. The Journal of Chemical Physics 101: 4177-4189.
27. Allen TW, Andersen OS, Roux B (2006) Molecular dynamics - potential of mean force calculations as a tool for understanding ion permeation and selectivity in narrow channels. Biophys Chem 124: 251-267.
28. Berneche S, Roux B (2001) Energetics of ion conduction through the K^+ channel. Nature 414: 73-77.
29. Shan Y, Klepeis JL, Eastwood MP, Dror RO, Shaw DE (2005) Gaussian split Ewald: A fast Ewald mesh method for molecular simulation. The Journal of chemical physics 122: 054101.
30. Tsushima RG, Li RA, Backx PH (1997) P-loop flexibility in Na+ channel pores revealed by single-and double-cysteine replacements. The Journal of general physiology 110: 59-72.
31. Kollman P (1993) Free energy calculations: applications to chemical and biochemical phenomena. Chemical reviews 93: 2395-2417.
32. Kumar S, Rosenberg JM, Bouzida D, Swendsen RH, Kollman PA (1992) The weighted histogram analysis method for free-energy calculations on biomolecules. I. The method. J Comput Chem 13: 1011-1021.

33. Liu P, Dehez Fo, Cai W, Chipot C (2012) A toolkit for the analysis of free-energy perturbation calculations. Journal of chemical theory and computation 8: 2606-2616.
34. Bennett CH (1976) Efficient estimation of free energy differences from Monte Carlo data. Journal of Computational Physics 22: 245-268.
35. Aksimentiev A, Schulten K (2005) Imaging α-hemolysin with molecular dynamics: ionic conductance, osmotic permeability, and the electrostatic potential map. Biophysical journal 88: 3745-3761.
36. Cohen J, Arkhipov A, Braun R, Schulten K (2006) Imaging the migration pathways for O 2, CO, NO, and Xe inside myoglobin. Biophysical journal 91: 1844-1857.
