# Supplementary figures and images for: Selective ion permeation involves complexation with carboxylates and lysine in a model human sodium channel
Source: PLoS Comput Biol. 2018 Sep 12;14(9):e1006398. doi: 10.1371/journal.pcbi.1006398 (PMC6152994; doi:10.1371/journal.pcbi.1006398)

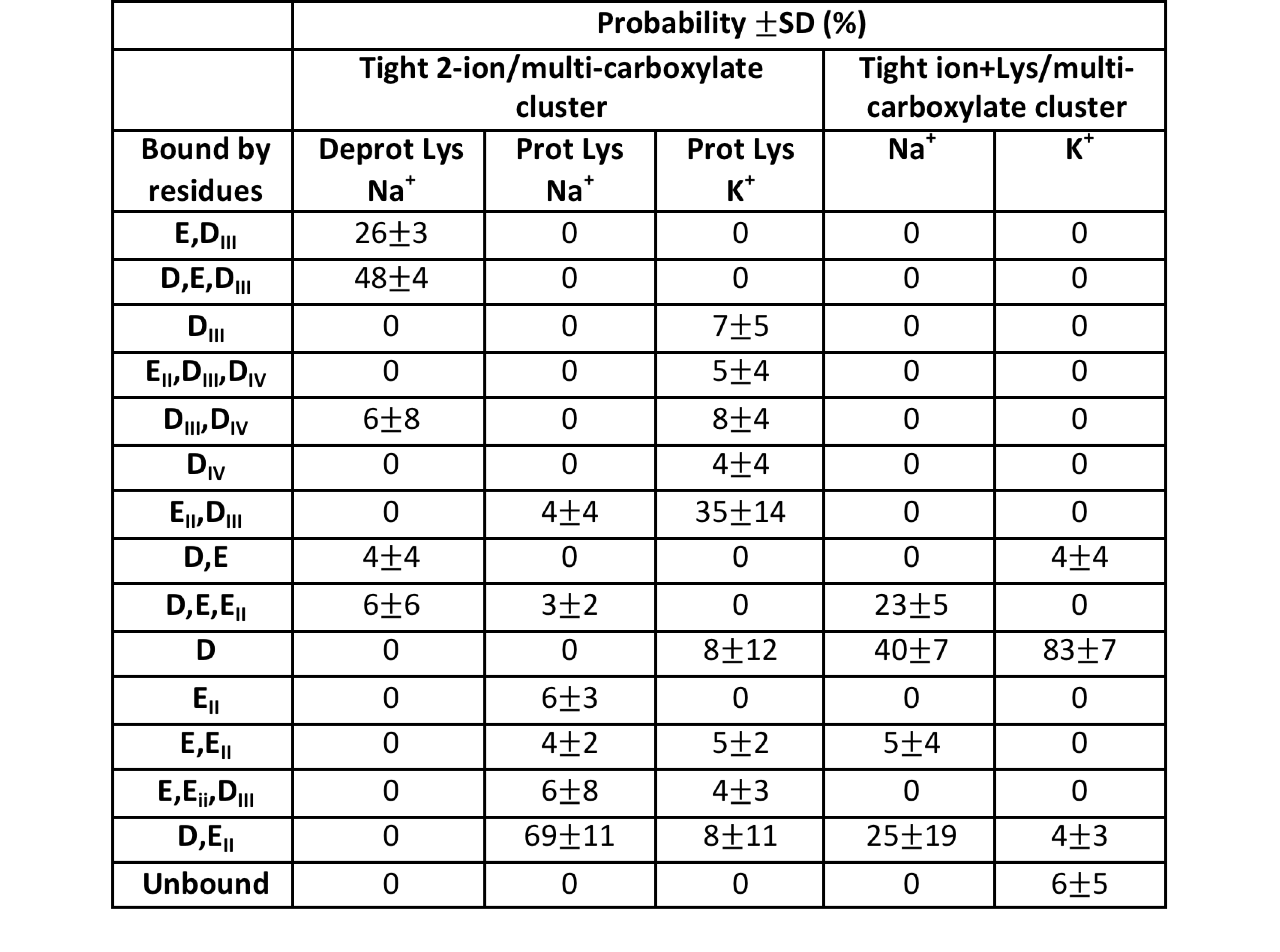

Supplement: S1 Table — The first column defines which carboxylate(s) the ion(s) are bound to. Columns 2,3 and 4 show the tight 2-ion/multi-carboxylate clusters for the Nav1.2 SF with deprotonated Lys0 in NaCl, protonated Lys+ in NaCl, and protonated Lys+ in KCl solutions, respectively. Columns 5 and 6 show the tight ion+Lys/multi-carboxylate clusters with protonated Lys+ in NaCl and KCl solutions, respectively. Clusters that occur less than 4% of the time are not shown. Most commonly, Na+ is bound to D and/or E together with EII, whereas K+ is bound to the outer EEDD ring or singly bound to only the DEKA D side chain. (TIFF) [file pcbi.1006398.s002.tiff]

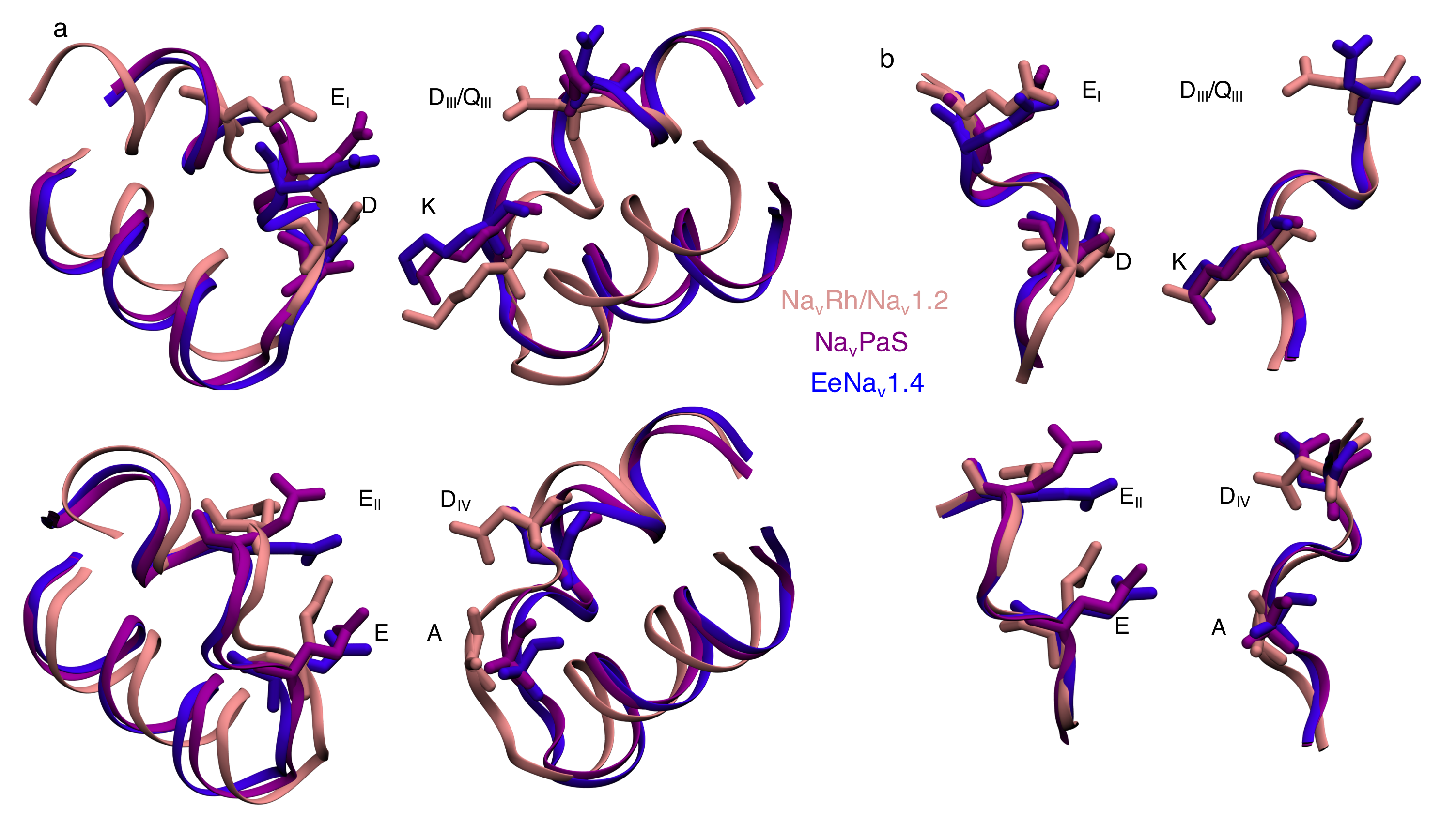

Supplement: S1 Fig — Alignments of model human Nav1.2 (pink) cockroach NavPaS (purple) and eel Nav1.4 (blue), showing DI and DIII on top and DII and DIV at the bottom. Panel a) shows the alignment according to the backbone of the SF and outer vestibule based on all four subunits, while b) shows alignment according to the SF and vestibule subunit by subunit, revealing the similarity of the Nav1.2 model to NavPaS and EeNav1.4 cryoEM structures. Side chains of Glu, Asp and Lys are indicated with sticks. (TIF) [file pcbi.1006398.s003.tif]

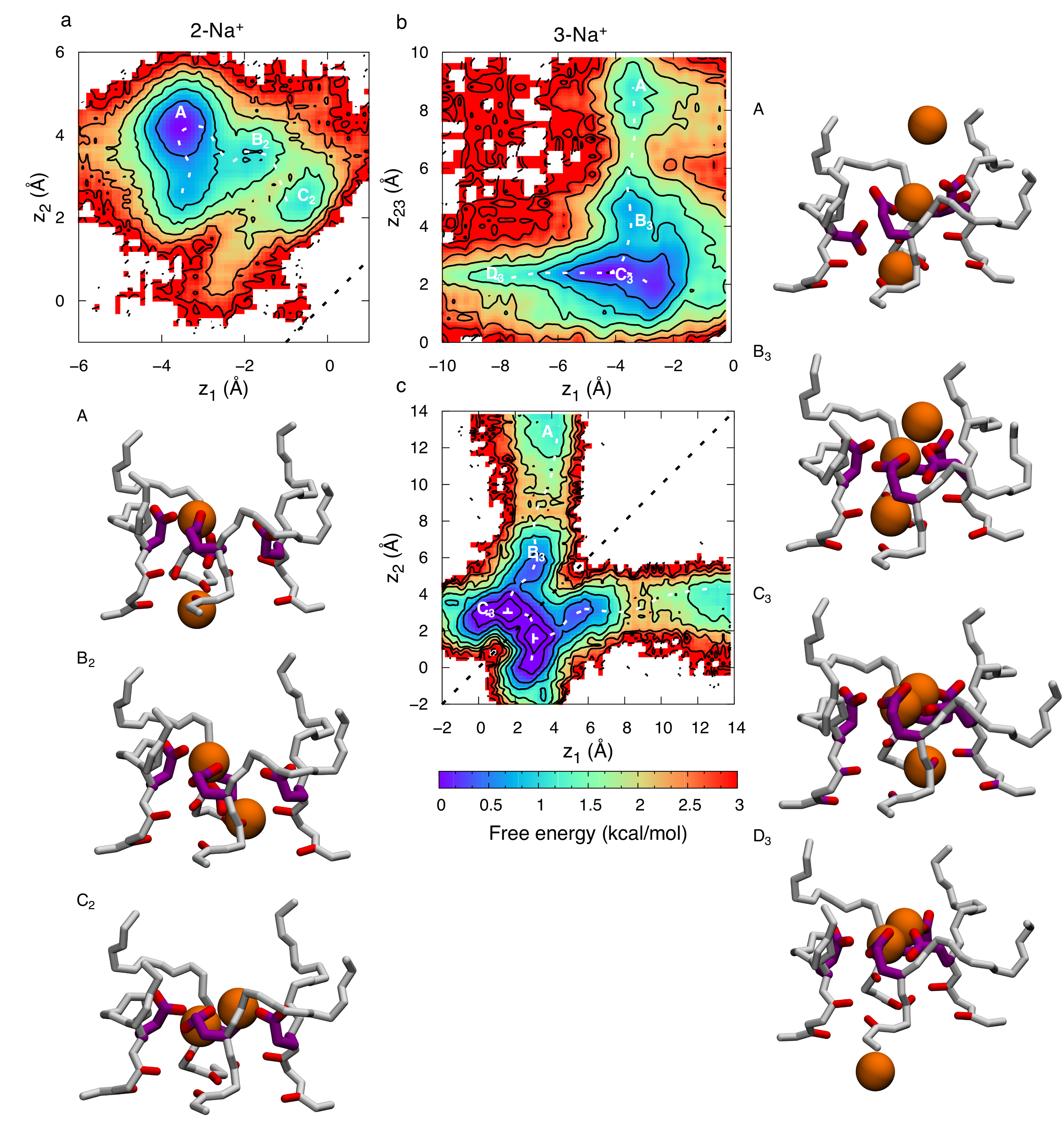

Supplement: S2 Fig — 2D free energy projections showing: a) 2-ion occupancy; and b & c) 3-ion occupancy (graphed for all three ions in b, and the top two ions in c); where z1, z2, and z3 correspond to the z positions of the bottom, middle and top ions, respectively, and where z23 is the z position of the COM of the top two ions. When there are 2 ions in the SF, the ions are trapped and it is not until a third ion enters from above that we can observe either a knock on or pass by conduction. Snapshots, with the EEEE ring in purple, indicate the corresponding Na+ ion (orange balls) movements. State labels include a subscript 2 or 3, representing the 2-ion or 3-ion mechanisms, respectively. Here the state label A has no subscript because A2 and A3 represent the same state, with same 2-ion configuration in the SF, but with approaching 3rd ion from bulk. (TIF) [file pcbi.1006398.s004.tif]

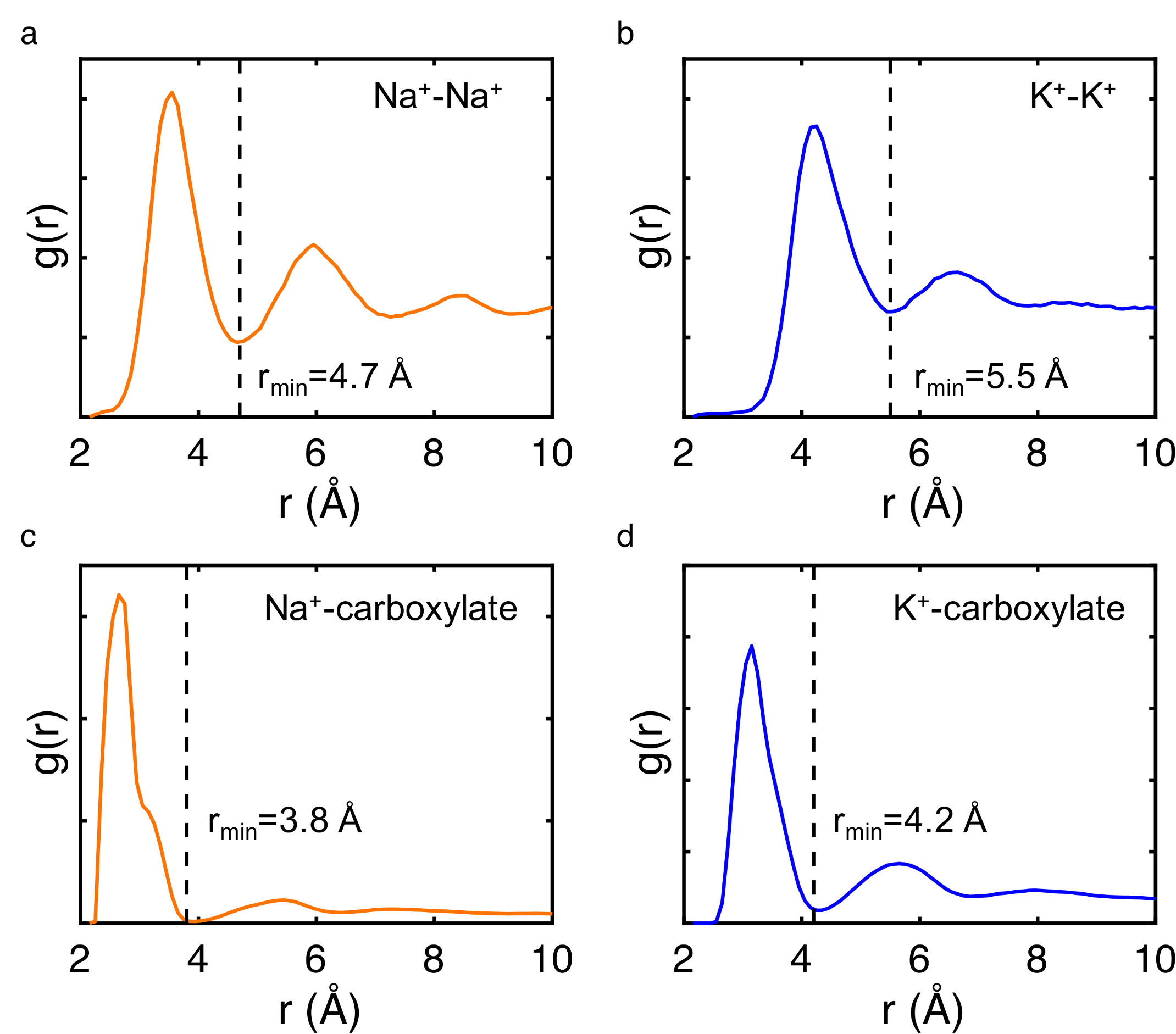

Supplement: S3 Fig — Distributions are shown for Na+-Na+ (a), K+-K+ (b), Na+-carboxylate (c) and K+-carboxylate (d). Multi-ion clusters defined by r < 4.7 Å for Na+ and r < 5.5 Å for K+, and carboxylate binding by r < 3.8 Å for Na+ and r < 4.2 Å for K+. (TIF) [file pcbi.1006398.s005.tif]

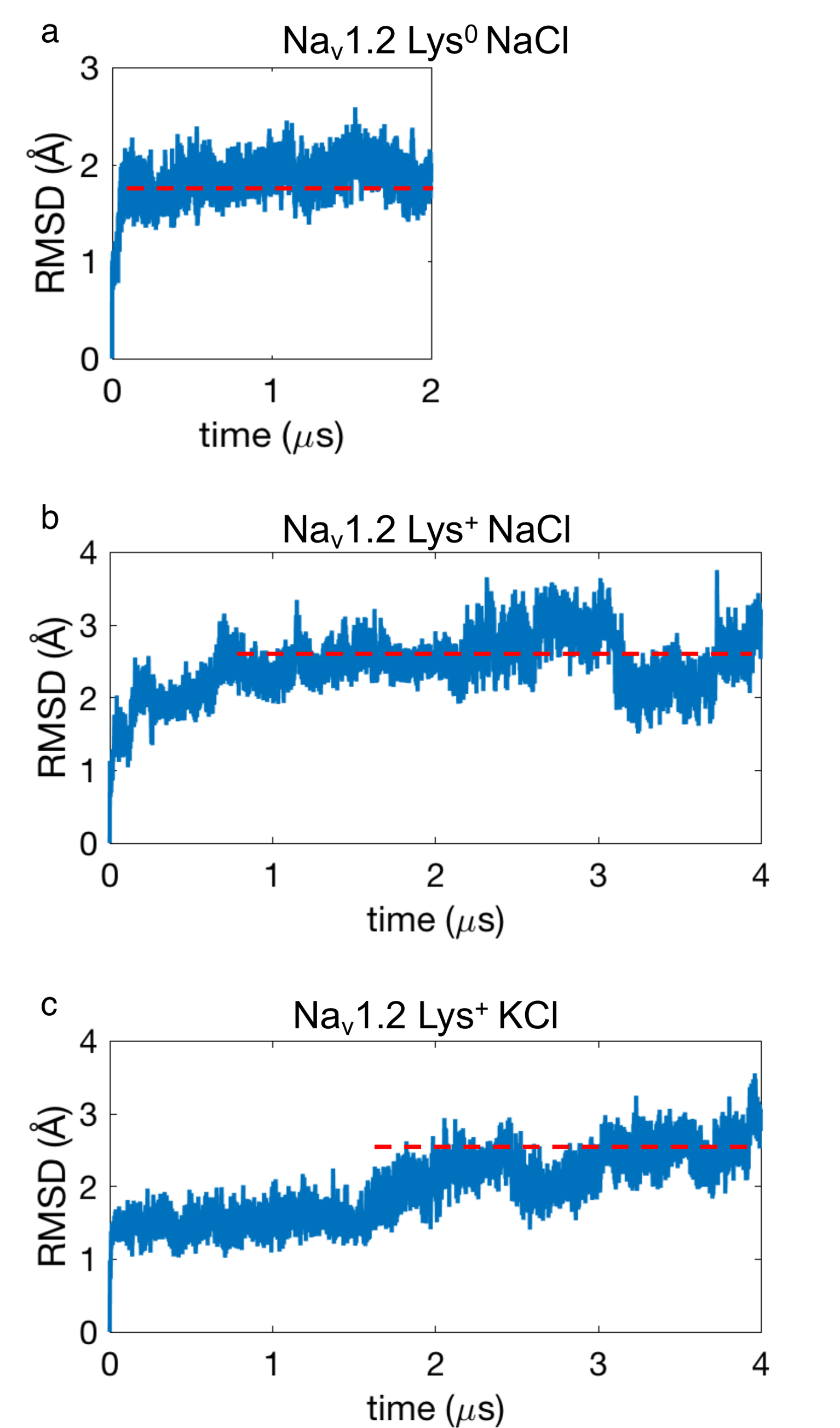

Supplement: S4 Fig — a) Na+ with deprotonated Lys0; b) Na+ with protonated Lys+; and c) K+ with protonated Lys+. Red dashed lines indicate the level of RMSD after the equilibration period. Late changes due to the onset of conduction, particularly for K+, are discussed in the text. (TIF) [file pcbi.1006398.s006.tif]

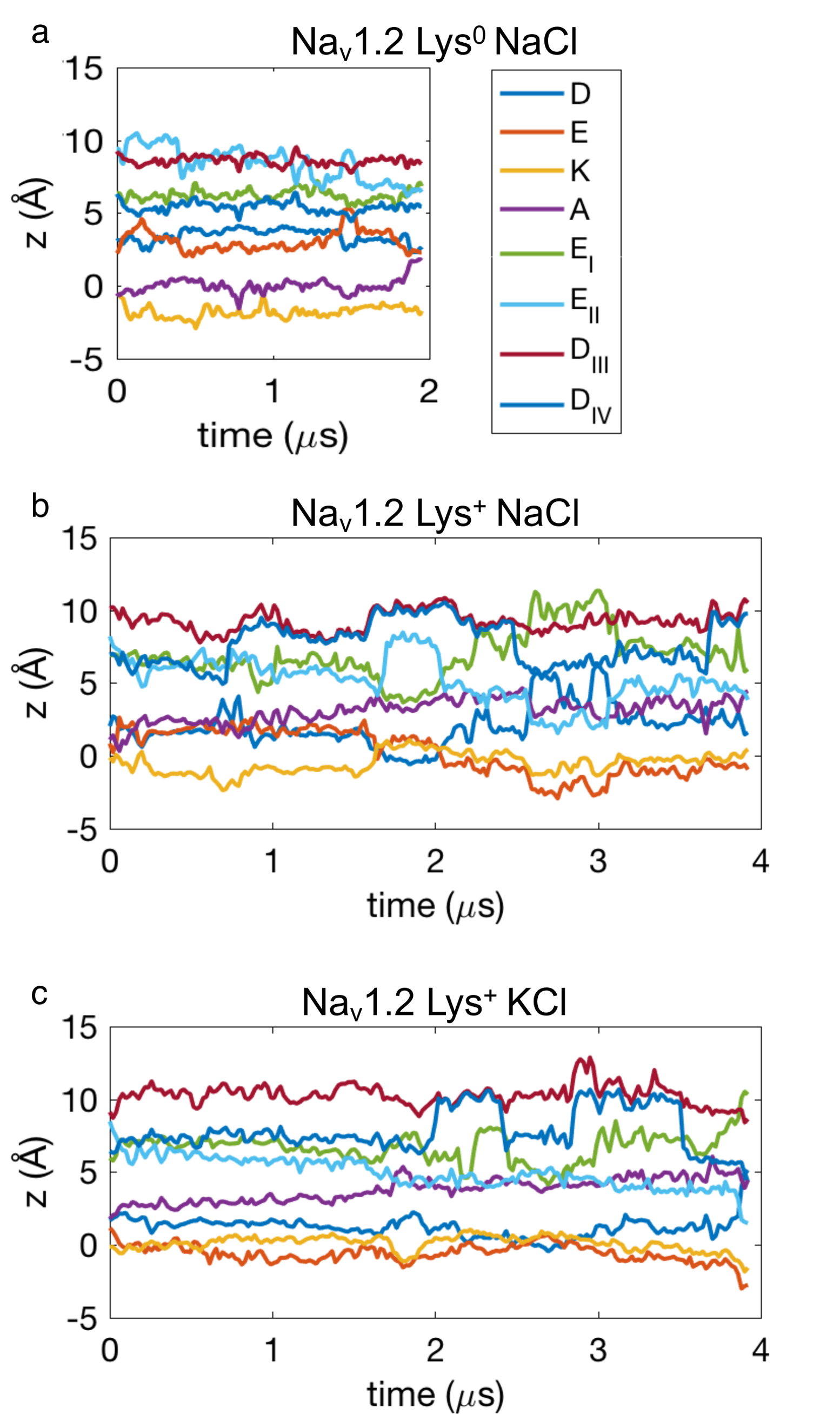

Supplement: S5 Fig — a) Na+ with deprotonated Lys0; b) Na+ with protonated Lys+; and c) K+ with protonated Lys+. (TIF) [file pcbi.1006398.s007.tif]

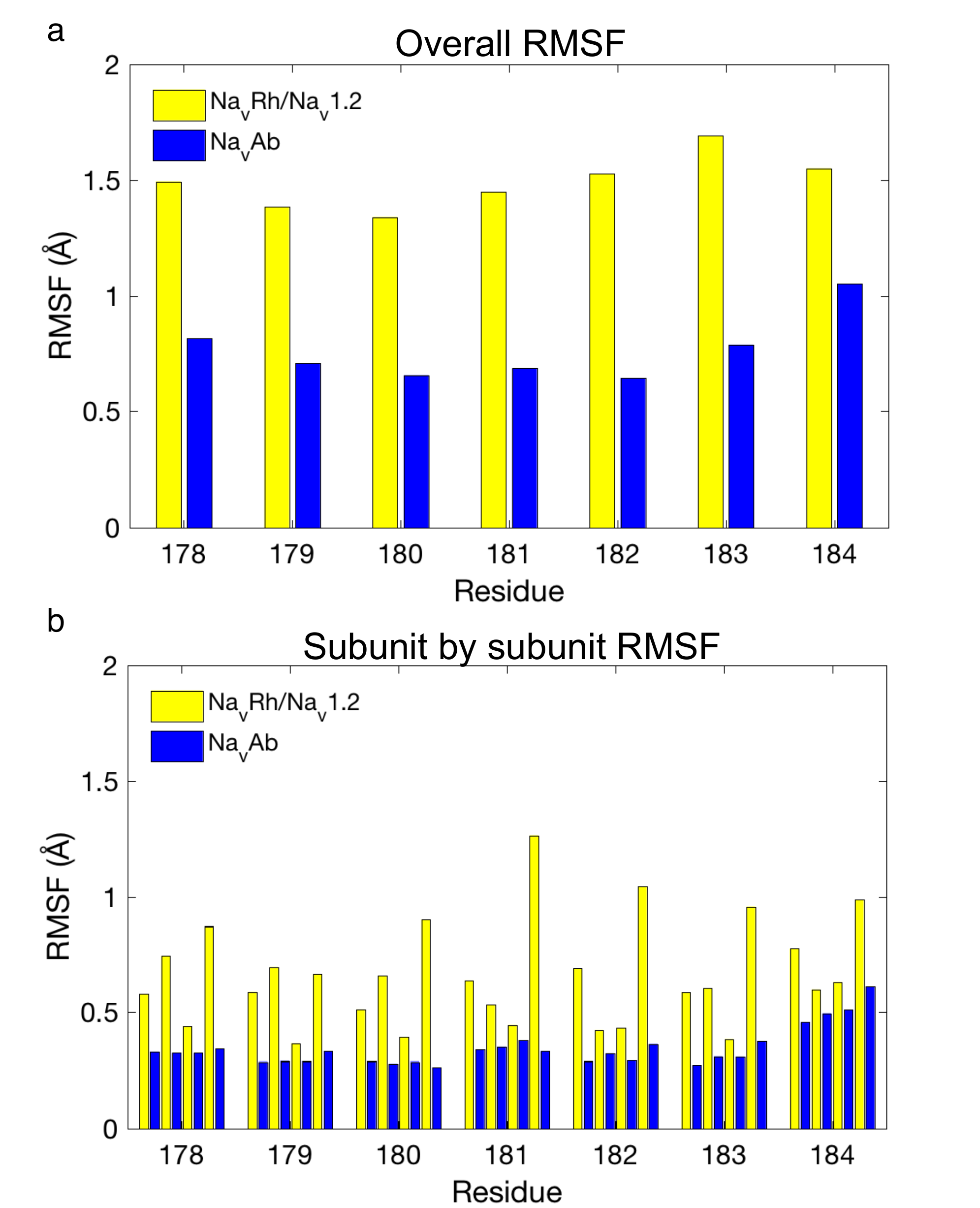

Supplement: S6 Fig — Fluctuations are shown for residues 178 to 184 in NavRh/Nav1.2 (yellow) and NavAb (blue) for: a) the mean of the four subunits (where orientation was according to all 4 subunits); and b) each individual subunit (where orientation was according to the subunit of interest). (TIF) [file pcbi.1006398.s008.tif]

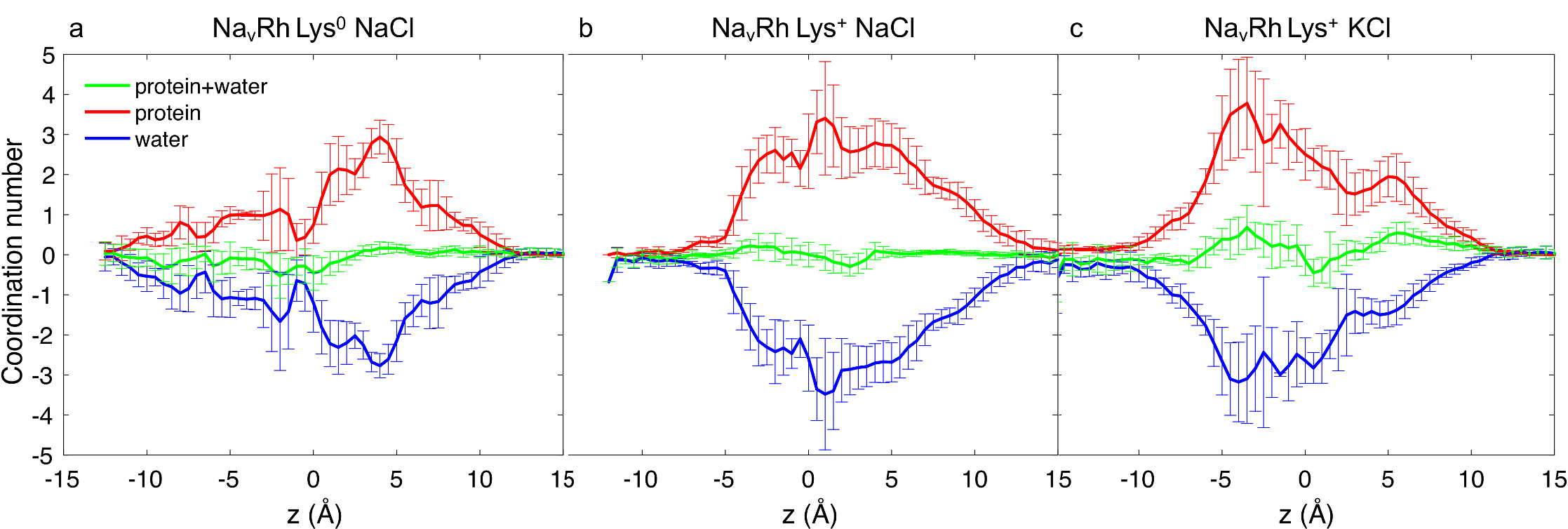

Supplement: S7 Fig — Mean ion coordination numbers relative to bulk water in NavRh/Nav1.2 for: a) Na+ with deprotonated Lys0; b) Na+ with protonated Lys+; and c) K+ with protonated Lys+. Bulk hydration numbers are 5.67±0.04 for Na+, and 6.94±0.07 for K+. (TIF) [file pcbi.1006398.s009.tif]

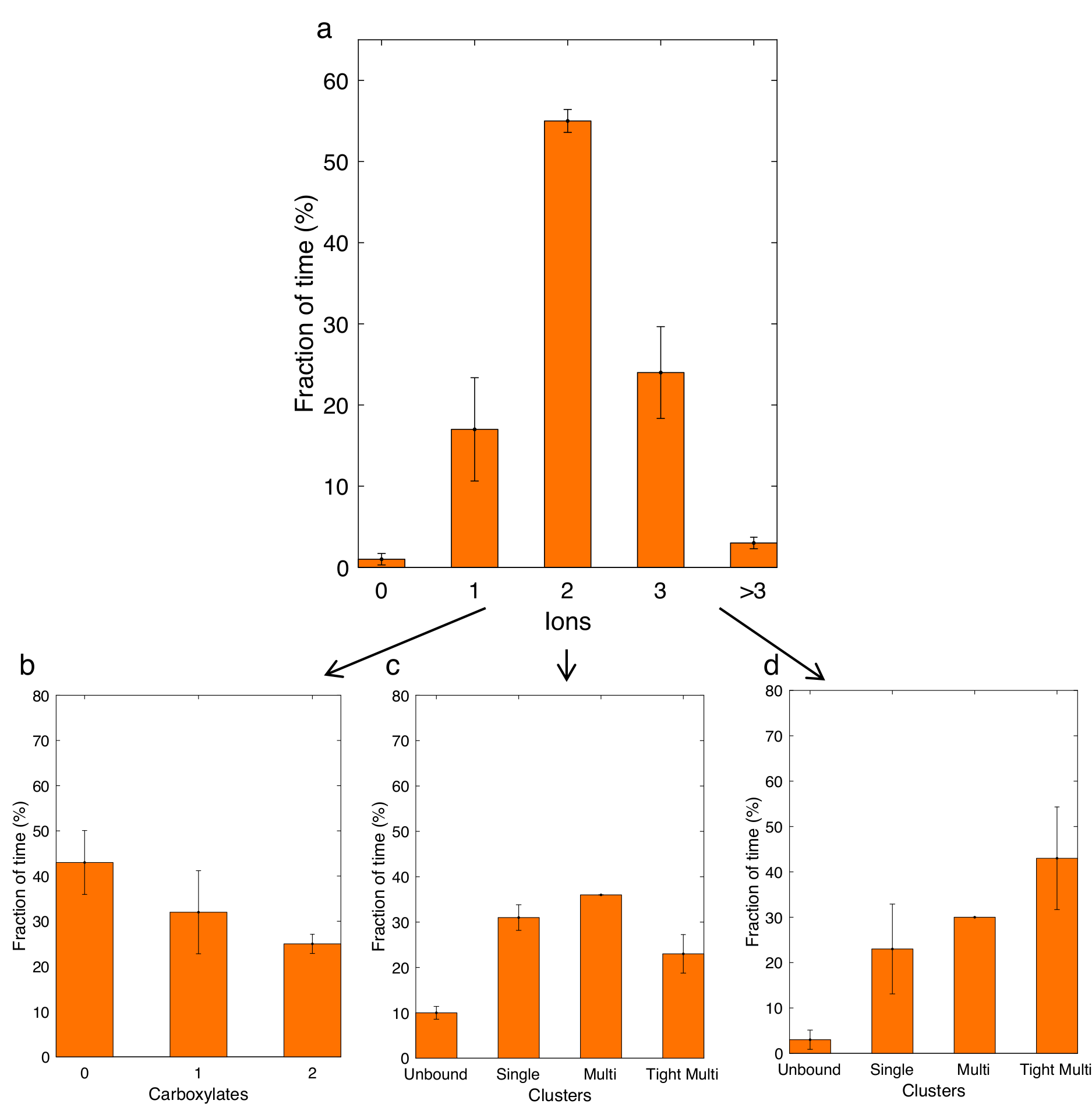

Supplement: S8 Fig — a) Distribution of Na+ occupancy and dominant clusters representing complexes with: b) 1-ion occupancies; c) 2 ion occupancies; and d) 3 ion occupancies. In panels c and d, ‘single’ clusters include 1-ion/1-carboxylate complexes only, 'multi' clusters include 1-ion/multi-carboxylate complexes with a loosely associated second ion, and ‘tight multi’ clusters represent 2-ion/multi-carboxylate clusters where both ions are within an ion-ion cut off, defined from the radial distribution function g(r) for ion-ion distance (see text). The channel range is defined by the region -15<z<15 Å. (TIF) [file pcbi.1006398.s010.tif]

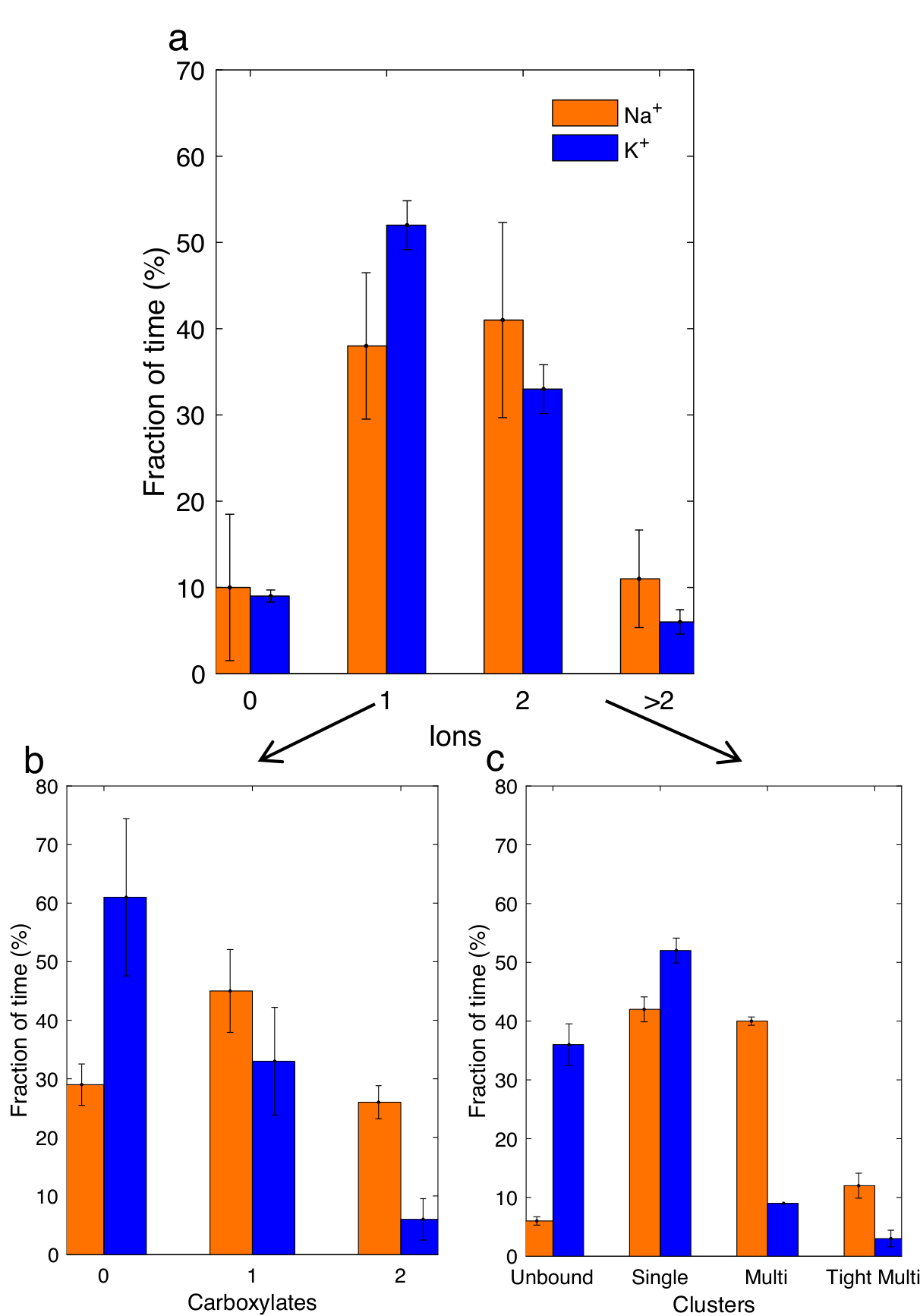

Supplement: S9 Fig — a) Distribution of ion occupancies. Panels b and c show dominant cluster conformations for: 1-ion (b) and 2 ion (c) occupancies, for Na+ (orange) and K+ (blue). See S8 Fig for definitions. (TIF) [file pcbi.1006398.s011.tif]

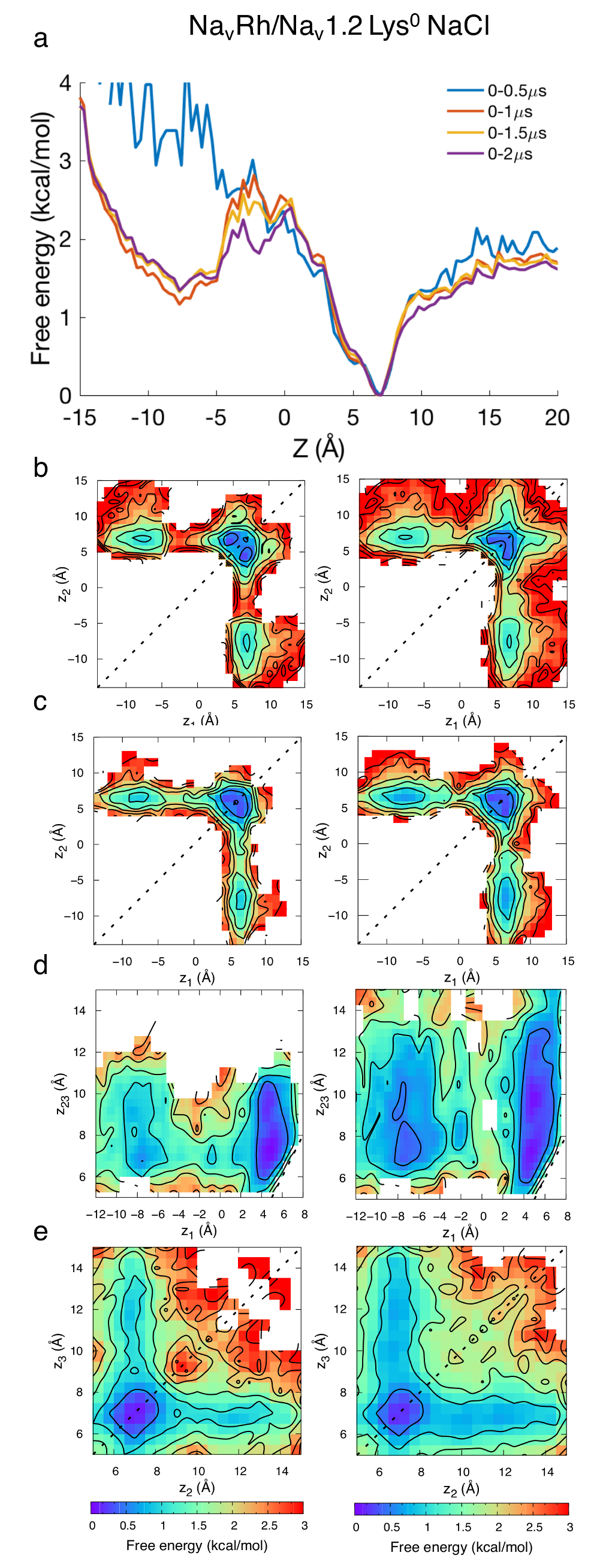

Supplement: S10 Fig — Panel a shows the convergence of 1D free energy for the ion across the channel, comparing time ranges specified in the legend. The free energy profile converges quickly in the outer vestibule and SF, but more slowly in the central cavity because it takes longer for ions to enter this part of the channel. Panels b-e show convergence of 2D free energy maps corresponding to Fig 4, for the last 2 μs of each simulation (left and right show time ranges 0–1 μs and 0–2 μs, respectively). (TIF) [file pcbi.1006398.s012.tif]

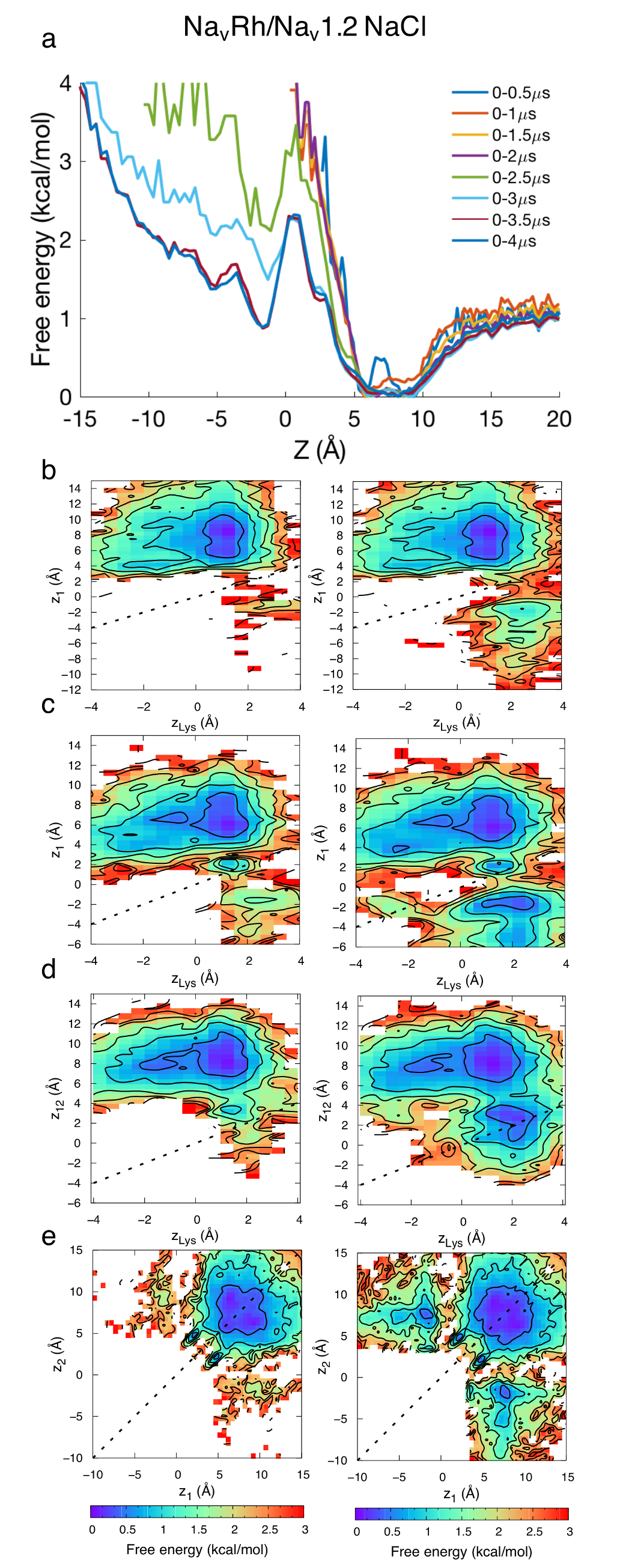

Supplement: S11 Fig — Panel a shows the convergence of 1D free energy for the ion across the channel, comparing time ranges specified in the legend. Panels b-e, show convergence of 2D maps corresponding to Figs 6 & 7 (left and right show time ranges 0–3 μs and 0–4 μs, respectively). (TIF) [file pcbi.1006398.s013.tif]

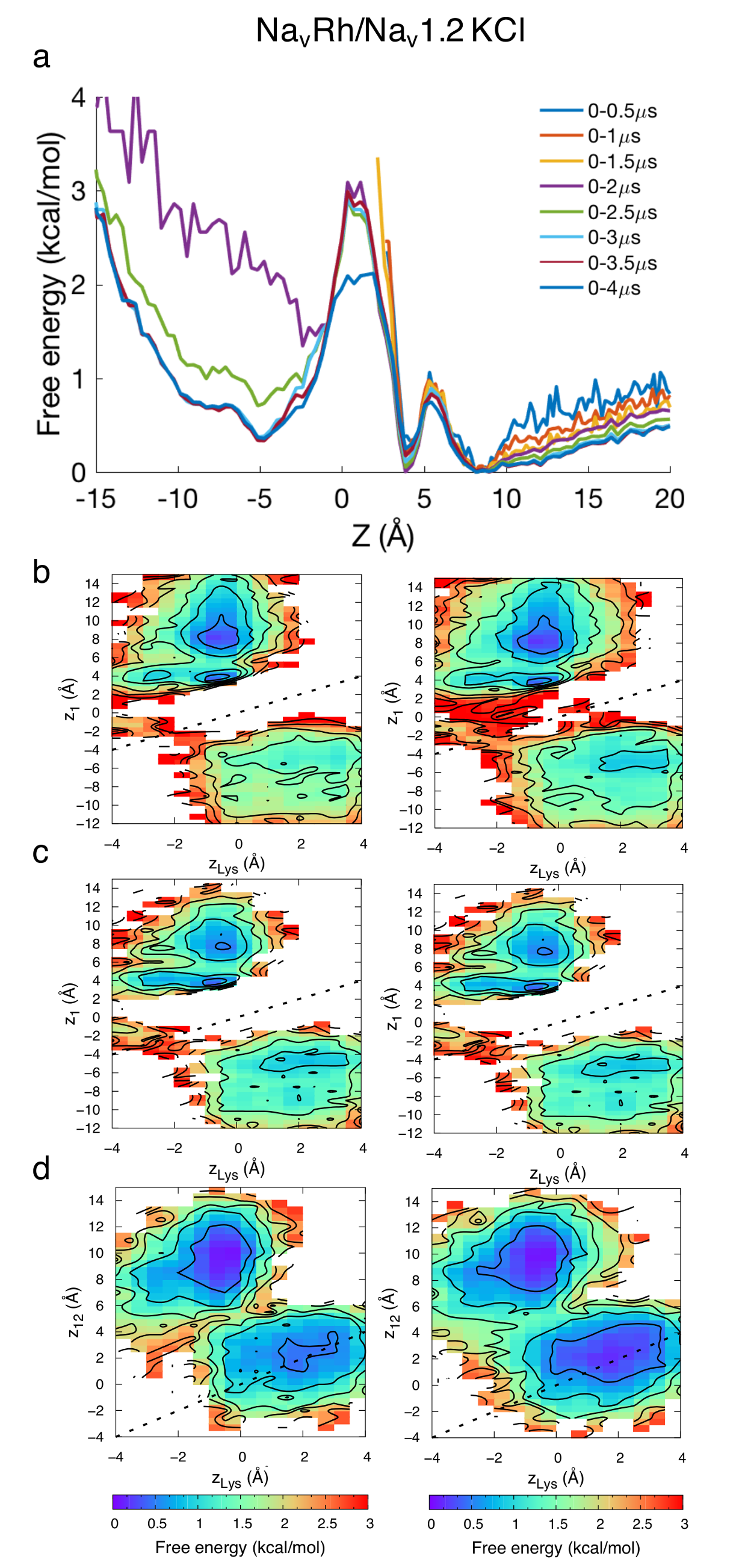

Supplement: S12 Fig — Panel a shows the convergence of 1D free energy for the ion across the channel, comparing time ranges specified in the legend. Panels b-d show convergence of 2D maps, corresponding to Fig 9 (left and right show time ranges 0–3 μs and 0–4 μs, respectively). (TIF) [file pcbi.1006398.s014.tif]
